# Supplementary material for: Effect of vitamin D supplementation in patients with chronic hepatitis C after direct-acting antiviral treatment: a randomized, double-blind, placebo-controlled trial
Source: PeerJ. 2021 Feb 9;9:e10709. doi: 10.7717/peerj.10709 (PMC7879942; doi:10.7717/peerj.10709)
Supplement: Supplemental Information 1 — (A) Baseline VD levels. (B) Sex. (C) Presence of cirrhosis. (D) Liver fibrosis severity by FIB4 score. (E) Liver fibrosis severity by APRI score. [file peerj-09-10709-s001.docx]

**Supplementary Table S1.** Subgroup analysis of changes in serum fibrogenesis markers by randomized arm

A. Baseline VD levels

| **Subgroup** | **N (VD/Placebo)** | **TGF-β1 (ng/mL)** | **TIMP-1 (ng/mL)** | **MMP-9 (ng/mL)** | **P3NP (ng/mL)** |
| --- | --- | --- | --- | --- | --- |
| VD <20 ng/mL | 20/30 | -0.2 (-2.5 – 2.2) | 5.6 (-24.0 – 35.2) | 94.0 (-103.1 – 291.1) | -2.3 (-4.8 – 0.23) |
| VD 20-30 ng/mg | 17/8 | 0.2 (-5.9 – 5.6) | -20.3 (-46.3 – 5.6) | 270.0 (-214.6 – 754.5) | 1.9 (-2.7 – 6.4) |
| P for interaction |  | 0.99 | 0.27 | 0.42 | 0.09 |

B. Sex

| **Subgroup** | **N (VD/Placebo)** | **TGF-β1 (ng/mL)** | **TIMP-1 (ng/mL)** | **MMP-9 (ng/mL)** | **P3NP (ng/mL)** |
| --- | --- | --- | --- | --- | --- |
| Males | 16/14 | -2.1 (-6.89 - 2.69) | -3.65 (-23.33 – 19.02) | 268.96 (-141.68 - 679.59) | -0.7 (-4.8 - 3.4) |
| Females | 21/24 | 0.54 (-1.74 - 2.83) | -4.46 (-35.75 - 26.83) | 28.19 (-155.91 - 212.3) | 0.13 (-2.6 - 2.86) |
| P for interaction |  | 0.27 | 0.97 | 0.23 | 0.72 |

C. Presence of cirrhosis

| **Subgroup** | **N (VD/Placebo)** | **TGF-β1 (ng/mL)** | **TIMP-1 (ng/mL)** | **MMP-9 (ng/mL)** | **P3NP (ng/mL)** |
| --- | --- | --- | --- | --- | --- |
| Cirrhosis | 21/24 | -0.13 (-2.28 - 2.01) | -17.58 (-45.33 - 10.16) | 105.35 (-109.04 - 319.74) | -0.45 (-3.42 - 2.52) |
| Non-cirrhosis | 16/14 | -0.96 (-5.9 - 3.98) | 12.98 (-20.06 - 46.01) | 167.71 (-206.79 - 542.21) | 0.39 (-3.39 - 4.17) |
| P for interaction |  | 0.73 | 0.16 | 0.75 | 0.72 |

| **Subgroup** | **N (VD/Placebo)** | **TGF-β1 (ng/mL)** | **TIMP-1 (ng/mL)** | **MMP-9 (ng/mL)** | **P3NP (ng/mL)** |
| --- | --- | --- | --- | --- | --- |
| FIB4 ≥3.25 | 9/14 | -0.35 (-2.55 - 1.85) | -34.81 (-78.18 - 8.55) | -36.07 (-245.31 - 173.17) | -1.51 (-5.79 - 2.77) |
| FIB4 <3.25 | 28/24 | -0.73 (-3.95 - 2.5) | 8.31 (-15.61 - 32.23) | 193.82 (-71.94 - 459.57) | 0.6 (-2.19 - 3.39) |
| P for interaction |  | 0.88 | 0.06 | 0.29 | 0.41 |

D. Liver fibrosis severity by FIB4 score

E. Liver fibrosis severity by APRI score

| **Subgroup** | **N  (VD/Placebo)** | **TGF-β1 (ng/mL)** | **TIMP-1 (ng/mL)** | **MMP-9 (ng/mL)** | **P3NP (ng/mL)** |
| --- | --- | --- | --- | --- | --- |
| APRI ≥1 | 7/9 | -1.95 (-3.66 - -0.24) | -34 (-94.67 - 26.67) | 32.63 (-240.76 - 306.03) | -1 (-5.98 - 3.97) |
| APRI <1 | 30/29 | -0.22 (-3.11 - 2.67) | 2.21 (-19.81 - 24.22) | 149.8 (-86.67 - 386.27) | 0.17 (-2.47 - 2.8) |
| P for interaction |  | 0.54 | 0.16 | 0.63 | 0.68 |
