# Supplementary material for: Effect of vitamin D supplementation in patients with chronic hepatitis C after direct-acting antiviral treatment: a randomized, double-blind, placebo-controlled trial
Source: PeerJ. 2021 Feb 9;9:e10709. doi: 10.7717/peerj.10709 (PMC7879942; doi:10.7717/peerj.10709)
Supplement: Supplemental Information 2 [file peerj-09-10709-s002.docx]

**Supplementary Table S2.** Changes in VD and serum fibrogenesis markers in patients with baseline VD <10 ng/mL

| Group | n | Changes at 6 weeks (ng/mL) | | | | |
| --- | --- | --- | --- | --- | --- | --- |
|  |  | **VD** | **TGF-β1** | **TIMP-1** | **MMP-9** | **P3NP** |
| Vitamin D | 3 | 33.1±17.7 | 8.8±3.3 | 110.1±94.2 | 419.6±897.7 | -1.0±2.5 |
| Placebo | 3 | 0.6±1.8 | 3.5±4.2 | 23.7±13.7 | -199.7±370.9 | 0.7±4.0 |
| p value |  | 0.034 | 0.155 | 0.188 | 0.331 | 0.572 |
