# Supplementary material for: Effect of vitamin D supplementation in patients with chronic hepatitis C after direct-acting antiviral treatment: a randomized, double-blind, placebo-controlled trial
Source: PeerJ. 2021 Feb 9;9:e10709. doi: 10.7717/peerj.10709 (PMC7879942; doi:10.7717/peerj.10709)
Supplement: Supplemental Information 4 [file peerj-09-10709-s004.pdf]

Related Manuscript (English)

Effect of vitamin D supplementation in patients with chronic hepatitis C after direct-acting antiviral treatment: a randomized, double-blind, placebo-controlled trial

Supachaya Sriphoosanaphan[1,2], Kessarin Thanapirom[1,2,3], Sirinporn Suksawatamnuay[1,2,3], Panarat Thaimai[1], Sukanya Sittisomwong[1], Kanokwan Sonsiri[1], Nunthiya Srisoonthorn[2], Nicha Teeratorn[1], Nattaporn Tanpowpong[4], Bundit Chaopathomkul[4], Sombat Treeprasertsuk[1], Yong Poovorawan[5], Piyawat Komolmit*[1,2,3]

1. Division of Gastroenterology, Department of Medicine, Faculty of Medicine, Chulalongkorn University and King Chulalongkorn Memorial Hospital, Bangkok, Thailand
2. Centre of Excellence in Liver Diseases, King Chulalongkorn Memorial Hospital, Bangkok, Thailand Thai Red Cross, Pathumwan, Bangkok Thailand
3. Liver Fibrosis and Cirrhosis Research Unit, Chulalongkorn University, Bangkok, Thailand
4. Department of Radiology, Faculty of Medicine Chulalongkorn University, King Chulalongkorn Memorial Hospital, Bangkok, Thailand
5. Centre of Excellence in Clinical Virology, Faculty of Medicine, Chulalongkorn University, Bangkok, Thailand

*Corresponding author: Piyawat Komolmit, MD, PhD.

Division of Gastroenterology, Department of Medicine, Faculty of Medicine, Chulalongkorn University, Bangkok 10330, Thailand Tel: +662-2564265, +669-47825195
Email: pkomolmit@yahoo.co.uk

Table of contents	Page
1. Research Protocol in English	2
2. Supplement documents	
a. Information for participant	15
b. Informed consent sheet	22
c. Case record form	25

Research Protocol

1. Proposal Title

Correction of vitamin D deficiency in chronic hepatitis C patients who had sustained virologic response after direct-acting agent therapy: Effect on serum hepatic fibrogenesis markers

2. Investigators

Dr. Piyawat Komolmit

Dr. Supachaya Sriphoosanaphan

Affiliation: Division of Gastroenterology and Hepatology

Department of Medicine, Faculty of Medicine

Chulalongkorn University

3. Background and rationale

Chronic hepatitis C is a common cause of chronic liver diseases, resulting in progressive cirrhosis and hepatocellular carcinoma [1,2]. Mechanisms behind hepatic fibrogenesis are active infection and inflammation which induce hepatic stellate cells (HSCs) to the activated myofibroblast like cells called activated HSCs. Fibrogenic cytokines responsible for these initial processes are transforming growth factor (TGF β_1), tissue inhibitor of matrix metalloproteinases (TIMP). Additionally, the synthesis of matrix metalloproteinases (MMPs), the most important fibrolytic enzymes, decreases in the injured liver. Therefore, activated HSCs proliferate and secrete more and more extracellular matrix (ECM) components and ultimately the liver reaches the state of cirrhosis [3].

Vitamin D (VD) is previously believed to be solely involved in calcium homeostasis. Nowadays, it has been known that VD acts as a hormone, autocrine and paracrine, involves in various kinds of body regulations. It involves in both innate and adaptive immunes responses and keeps balance of the T and B cells ‘cytokines [4].

Degree of VD deficiency is worsening in CHC patients with progressive liver cirrhosis [5-7]. Several studies demonstrated that chronic hepatitis C (CHC) patients with VD deficiency had lower sustained virological response (SVR) than the patients with normal VD levels when treated by interferon/ribavirin regimens. In addition, VD supplement help to reduce inflammation and improve SVR [8,9]. Recent data suggest that VD inhibits HSCs proliferation and hepatic fibrogenesis [10,11].

Our previous study demonstrated that restoration of VD deficiency in patients with CHC improves the serum fibrogenesis markers. There is a change toward fibrolytic activities [12]. This evidence highlights the role of VD in human hepatic fibrogenesis.

The possible explanations of protective role of VD in liver fibrosis could be due to three main mechanisms.

1. HCV viral replication
2. Inflammation reduction
3. Direct effect on hepatic fibrogenesis

However, little is still known about the exact properties of VD on hepatic fibrogenesis. In this study, we aim to explore and clarify the exact role of VD. We study the patients with CHC who underwent curative direct-acting antivirals (DAA) treatment. We believe that these patients do not have the viral replication and hepatic inflammation left. Therefore, we hypothesized that restoration of VD deficiency after curative treatment would further attenuate liver fibrosis, as assessed by the improvement of fibrogenesis markers; TGF-β_1, TIMPs, MMPs, and P3NP.

4. Review of related literatures

Viral hepatitis C is an important cause of chronic liver diseases, cirrhosis and hepatocellular carcinoma. Initial phase of HCV infection, host innate immune responses involve in controlling of infection by natural killer cells (NK cells), plasmacytoid dendritic cells (pDC) and monocytes. Subsequently adaptive immune responses take a role during chronic infection phase which several cells including cytotoxic T cell (CD8) and several cytokines involve and result in chronic inflammation and fibrogenesis in the liver[1,13].

Hepatic fibrogenesis

Chronic inflammation of the livers caused by several diseases including chronic viral hepatitis B and C, drugs and toxins, alcohol, autoimmune, non-alcoholic fatty liver, and others have important

role in hepatic fibrogenesis. Chronic induction by inflammatory cytokines induces hepatic stellate cells (HSCs), from a quiescent cell to a myofibroblast like cell, called activated HSC, and initiate fibrotic processes[3,14].

The quiescent HSCs normally excrete collagen type IV which is a component of basement membrane. After activated by specific cytokines, there are two subsequent phases, proliferation and perpetuation of the HSCs.

Mechanism and function of TGF β_1

TGF β_1 involves in several body functions, controlling ECM components and several diseases processes15. In mammal, there are three types of TGF β_1, called TGF β_1, TGF β_2 and TGF $\beta_{3..}$ TGF β_1 initially secreted from the cells as a latent TGF β_1 which composes of TGF β dimer and latency-associated peptide (LAP). This LAP is bonded to the latent TGF β binding protein (LTBP) which is attached to the ECM components. Upon enzymatic degradation of the LAP, the TGF β_1 becomes activated.

Association of TGF β_1 and hepatic fibrogenesis in CHC

A study in a CCl4 rat model of cirrhosis demonstrates the accumulation of collagens and increase levels of mRNA expression of TGF β_1 in the liver tissues[16]. Moreover, the serum levels of TGF β_1 were found to decrease in CHC patients who responded to interferon/ribavirin treatment [16-17]. To date there are several studies used TGF β_1 as a marker for hepatic fibrosis and it correlates with the degree of fibrosis. Increase in serum TGF β_1 levels is well-correlated with hepatic fibrosis as assessed by Metavir score ≥ F2 (AURIC = 0.85) and levels over 115 ng/mL was associated with rapid fibrotic progression [16,19-20].

VD and immune regulation

Previously, VD is thought to be involved in only body calcium homeostasis. Nowadays, VD is known to functions as a hormone, autocrine or paracrine. Importantly, VD involves in immune regulation both innate and adaptive immune responses as the active form of VD is locally generate and activate immune cells via the VDR which is expressed in cells of immune system. VD help to keep the balance of adaptive immune responses toward increase TH1 and reduction of TH2 cells and cytokines[4].

VD deficiency in patients with CHC

VD deficiency in patients with chronic hepatitis and cirrhosis is a common problem and is more prevalence than healthy individual. The reasons of deficiency might be the decrease in function of hepatic metabolism of VD and it is worsening along with the degree of liver dysfunction[21]. Other explanation may be from malnutrition, lack of UVB exposure due to the illness[22].

CHC patients have more VD deficiency than normal population and degree of deficiency increase with more severe hepatic fibrosis[22]. CHC patients with VD deficiency have more progressive fibrosis as compare to the patients without VD deficiency[23]. An in vitro study suggested that VD can inhibit HCV viral replication and suppressed inflammatory cytokines[25].

All in all, data suggested that VD deficiency is related to hepatic fibrogenesis. VD supplements, apart from improve general health, help to increase CHC treatment responses. We hypothesize that VD supplement might help to delay or improve hepatic fibrosis by correct the imbalance of profibrotic and pro-fibrolytic cytokines/enzymes involved in hepatic fibrogenesis. It is interesting to find out that VD supplement in patients with chronic hepatitis will change or reverse serum markers of fibrogenesis to pro-fibrolytic side even in patients with CHC after curative treatment with DAA.

5. Objectives

Primary objective

To study the effect of 6-week supplementation of VD on the changes of fibrogenic cytokine, TGF β_1 as compared to placebo

Secondary objective

To study the effect of 6-week supplement of VD on the changes of fibrolytic enzymes, TIMP-1, MMP-9, and P3NP as compared to placebo

6. Hypothesis

Primary research question:

Could restoration of VD levels in patients with CHC change the serum levels of fibrogenic cytokine, TGF β_1?

Secondary research question:

Could restoration of VD levels in CHC patients change the serum levels of TIMP-1, MMP-9, and P3NP?

7. Keywords

- TGF-β_1
- Vitamin D Supplement
- Chronic Hepatitis C
- Liver fibrosis

8. Research design

Randomized, double-blind, placebo controlled trial

9. Research methodology

Target population: Patients with CHC who underwent curative treatment with DAA within 1 year in Hepatology Clinic, King Chulalongkorn Memorial Hospital

Inclusion criteria

1. Patients with CHC who underwent curative treatment with DAA within 1 year
2. Age > 18 years old
3. Serum 25 (OH)VD < 30 ng/mL
4. Evidence of liver fibrosis after DAA
   a. Fibroscan (Transient elastogram > 7.1 kPa)
   b. Magnetic resonance elastography (MRE)
   c. Ultrasound elatography

Exclusion cirteria

1. Patients with these categories: autoimmune diseases, ischemic heart diseases, asthma, COPD, DM and malignancies

2. Patients diagnosed with other causes of chronic liver diseases eg. chronic hepatitis B, autoimmune hepatitis, nonalcoholic steatohepatitis, alcoholic liver diseases
3. Pregnancy and during breast feeding
4. During active bacterial or viral infections
5. During on treatment with steroids or immunosuppressive drugs or stopped those drugs in less than 6 months
6. Patients who had interferon within 12 months
7. Active alcoholic drinking over 20 grams per day or abstinence less than 6 months
8. Patients with chronic renal insufficiency (GFR less than 60 mL/minute)
9. Patients who currently on VD supplementation

Intervention

1. Explain the objective to the patients
2. Taking history and physical examination and recording CRF
3. Blood collection and screening for VD levels and including patients who had VD levels < 30 ng/mL
4. Randomization using stratification block randomization which generate the sequences by computer software. Associated staffs who not involve in the trial do this process and give the random sequence of each patients. Assign the patients to A or B groups and received the supplements.
5. Measuring of blood levels of VD, TGF-β_1, TIMP-1, MMP-9, and P3NP at baseline and 6 weeks
6. Statistics analysis of all parameters
7. Supplement VD to all patients in placebo group after the end of the study
8. Protocol for VD supplementation as shown in Table 1.

Diagnosis	Vitamin D level (ng /mL)	Replacement Total dose (IU/week)	Ergocalciferol (D2) 20,000 IU/ tab	Duration
Optimal	>30	-	-	-
Mild deficiency (insufficiency)	20–30	60,000	2 tabs Monday and 1 tab Friday	6 weeks
Moderate deficiency	10–20	80,000	2 tabs Monday and 2 tab Friday	6 weeks
Severe deficiency	<10	100,000	3 tabs Monday and 2 tabs Friday	6 weeks

Table 1. VD supplementation protocol

Blood collection of the TGF β_1 and other enzymes

1. Blood collection as a clotted blood (30 minutes stand)
2. Centrifugation for 15 minutes
3. Separate the serum and stored in -70C until the measurements
4. ELISA method (R&D system) for TGF β_1: use coefficient of variation of the intra assay 1.9 – 2.9% and inter assay 6.4 – 9.3%. Method of ELISA assay are performed according to the recommendation by R&D systems.

Blood test for 25 (OH)VD

1. Clotted bloods are collected
2. VD levels are measure using immuno-chemiluminescence assay

Sample size determination

$$\text{N per group} = \frac{2\,(Z_{1-\alpha/2} + Z_{1-\beta})^2\,\sigma^2}{(X_1 - X_2)^2}$$

The calculation based on a study by Kolmolmit P et al[12]. which measured a change of serum TGF β_1 in CHC patients before and after VD or placebo supplementation

$\alpha = 0.05 \rightarrow Z_{\alpha/2} = 1.96$

$\beta = 0.05 \rightarrow Z_{\beta} = 1.64$

Sample size in each group = 29, both groups = 58

Approximate dropout rate = 10%

Each group should have at least 32 cases and total number is 64 cases

10. Data collection

Baseline characteristics include age, genders, underlying diseases, history of alcoholic drinking, hepatitis C viral load, VD levels, LFTs, CBC, medications, side effects. All data are recorded in the CRF and computer using SPSS software.

11. Data analysis and statistics

Quantitative data

Comparing between two dependent data (pre- and post-VD supplements) are tested using paired t-tests and the independent data (VD group and placebo group) are tested by independent t-test or Mann-Whitney U test according to the distribution of the data and presented in mean, median and standard deviation.

Qualitative data

be presented in frequency and percentage, and use statistics of Chi-square test or Fisher's exact test per type of data

12. Ethic consideration

This trial is performed under Belmont Report's 3 basic principle for clinical study including respect for persons, beneficence, and justice.

Respect for person

Principal investigator and the team will not disclose any data related to personal data. Only principal investigator could access the study data. Participants in this trial will receive information regarding the study in detail of objective, benefits and side effects that might happen. The study could be done only after having informed consent.

Beneficial/non-maleficence

This study has benefit for the participants who had vitamin D deficiency and may help increase benefit of the hepatitis C treatment in the future. Vitamin D may help and expand the scientific knowledge of vitamin D involving in decrease inflammation and fibrogenesis. The participants may

have minor risk from drawing blood during the study (twice in 6 weeks), which we solve this by using experience nurse or staff taking blood test for the participants.

Justice

This study has clear criteria of inclusion and exclusion for selecting the patients who will be enrolled to study. This study is a randomized controlled trial which means all participants will have equal chance of risk and benefit. The patients who had placebo will at the end receive VD supplement after the trial completed.

13. Expected benefit and applications

Apart from VD supplement for patients who have VD deficiency, it is for the scientific knowledge of VD supplement that might help delaying the fibrotic outcome of chronic liver diseases and to support the results of in vitro and animal models previously reported. Moreover, the results could further clarify the role of VD on hepatic fibrosis amelioration.

14. Obstacles and strategies to solve the problems

As this trial aim to enroll a large number of patients. Patients might not be enough for the study in both arms and it is possible that some participants will loss follow up during the operation period. We have plan to solve these problems by giving information to the doctors and patients in the liver clinic. In addition, during the trial we will give explanation and follow up by phone regularly.

15. Timeline

One year

16. Venue of the study

Division of Gastroenterology and Hepatology, Department of Medicine, Faculty of Medicine, Chulalongkorn University

17. Tabulation of Research Activities and Timeline

Activity	2017					2018									
	8	9	10	11	12	1	2	3	4	5	6	7	8	9	10
1. Proposal and IRB															
2. Study period															
3. Data analysis															
4. Conclusion															

18. Budget

Items	Budget
1. Analysis for TGF-β_1, TIMP-1, MMP-9, P3NP<br>2. Ergocalciferol<br>3. Placebo<br>4. Blood collection	200,000<br>10,000<br>3,000<br>80,000
**Total**	**293,000**

19. References

1. Lauer GM, Walker BD. Hepatitis C virus infection. The New England journal of medicine. 2001;345(1):41-52. Epub 2001/07/07.
2. Shiffman ML. Natural history and risk factors for progression of hepatitis C virus disease and development of hepatocellular cancer before liver transplantation. Liver transplantation: official publication of the American Association for the Study of Liver Diseases and the International Liver Transplantation Society. 2003;9(11):S14-20. Epub 2003/10/31.
3. Bansal MB FS. Sherlock's Disease of the Liver and Biliary System 2011.
4. Di Rosa M, Malaguarnera M, Nicoletti F, Malaguarnera L. Vitamin D3: a helpful immuno-modulator. Immunology. 2011;134(2):123-39. Epub 2011/09/08.
5. Rahman AH, Branch AD. Vitamin D for your patients with chronic hepatitis C? Journal of hepatology. 2013;58(1):184-9. Epub 2012/08/09.
6. Petta S, Camma C, Scazzone C, Tripodo C, Di Marco V, Bono A, et al. Low vitamin D serum level is related to severe fibrosis and low responsiveness to interferon-based therapy in genotype 1 chronic hepatitis C. Hepatology. 2010;51(4):1158-67. Epub 2010/02/18.
7. Terrier B, Carrat F, Geri G, Pol S, Piroth L, Halfon P, et al. Low 25-OH vitamin D serum levels correlate with severe fibrosis in HIV-HCV co-infected patients with chronic hepatitis. Journal of hepatology. 2011;55(4):756-61. Epub 2011/02/22.
8. Abu-Mouch S, Fireman Z, Jarchovsky J, Zeina AR, Assy N. Vitamin D supplementation improves sustained virologic response in chronic hepatitis C (genotype 1)-naive patients. World journal of gastroenterology: WJG. 2011;17(47):5184-90. Epub 2012/01/05.
9. Nimer A, Mouch A. Vitamin D improves viral response in hepatitis C genotype 2-3 naive patients. World Journal of Gastroenterology : WJG. 2012;18(8):800-5. Epub 2012/03/01.
10. Abramovitch S, Dahan-Bachar L, Sharvit E, Weisman Y, Ben Tov A, Brazowski E, et al. Vitamin D inhibits proliferation and profibrotic marker expression in hepatic stellate cells and decreases thioacetamide-induced liver fibrosis in rats. Gut. 2011;60(12):1728-37. Epub 2011/08/06.
11. Potter JJ, Liu X, Koteish A, Mezey E. 1,25-dihydroxyvitamin D3 and its nuclear receptor repress human alpha1 (I) collagen expression and type I collagen formation. Liver international : official journal of the International Association for the Study of the Liver. 2013;33(5):677-86. Epub 2013/02/19.
12. Komolmit P, Kimtrakool S, Suksawatamnuay S, Thanapirom K, Chattrasophon K, et al. Vitamin D supplementation improves serum markers associated with hepatic fibrogenesis in chronic hepatitis C patients: A randomized, double-blind, placebo-controlled study. Sci Rep. 2017 Aug 21;7(1):8905
13. Spengler U, Nattermann J. Immunopathogenesis in hepatitis C virus cirrhosis. Clin Sci (Lond). 2007;112(3):141-55. Epub 2007/01/04.

14. Bataller R, Brenner DA. Liver fibrosis. The Journal of clinical investigation. 2005;115(2):209-18. Epub 2005/02/04.
15. Blobe GC, Schiemann WP, Lodish HF. Role of transforming growth factor beta in human disease. The New England journal of medicine. 2000;342(18):1350-8. Epub 2000/05/04.
16. Kanzler S, Baumann M, Schirmacher P, Dries V, Bayer E, Gerken G, et al. Prediction of progressive liver fibrosis in hepatitis C infection by serum and tissue levels of transforming growth factor-beta. Journal of viral hepatitis. 2001;8(6):430-7. Epub 2001/11/13.
17. Tsushima H, Kawata S, Tamura S, Ito N, Shirai Y, Kiso S, et al. Reduced plasma transforming growth factor-beta1 levels in patients with chronic hepatitis C after interferon-alpha therapy: association with regression of hepatic fibrosis. Journal of hepatology. 1999;30(1):1-7. Epub 1999/02/02.
18. Flisiak R JJ, Lapinski TW, Flisiak I, Prokopowiczi D. Effect of pegylated interferon alpha 2b plus ribavirin treatment on plasma transforming growth factor-b1, metalloproteinase-1, and tissue metalloproteinase inhibitor-1 in patients with chronic hepatitis C. WJG. 2005;11(43):6833-8.
19. Liu T, Wang X, Karsdal MA, Leeming DJ, Genovese F. Molecular serum markers of liver fibrosis. Biomarker insights. 2012;7:105-17. Epub 2012/08/09.
20. Martinez SM, Crespo G, Navasa M, Forns X. Noninvasive assessment of liver fibrosis. Hepatology. 2011;53(1):325-35. Epub 2011/01/22.
21. Cholongitas E, Theocharidou E, Goulis J, Tsochatzis E, Akriviadis E, Burroughs K. Review article: the extra-skeletal effects of vitamin D in chronic hepatitis C infection. Alimentary pharmacology & therapeutics. 2012;35(6):634-46. Epub 2012/02/10.
22. Lange CM, Bojunga J, Ramos-Lopez E, von Wagner M, Hassler A, Vermehren J, et al. Vitamin D deficiency and a CYP27B1-1260 promoter polymorphism are associated with chronic hepatitis C and poor response to interferon-alfa based therapy. Journal of hepatology. 2011;54(5):887-93. Epub 2010/12/15.
23. Baur K, Mertens JC, Schmitt J, Iwata R, Stieger B, Eloranta JJ, et al. Combined effect of 25-OH vitamin D plasma levels and genetic vitamin D receptor (NR 1I1) variants on fibrosis progression rate in HCV patients. Liver international : official journal of the International Association for the Study of the Liver. 2012;32(4):635-43. Epub 2011/12/14.
24. Autier P, Gandini S. Vitamin D supplementation and total mortality: a meta-analysis of randomized controlled trials. Archives of internal medicine. 2007;167(16):1730-7. Epub 2007/09/12.
25. Kitson MT, Roberts SK. D-livering the message: the importance of vitamin D status in chronic liver disease. Journal of hepatology. 2012;57(4):897-909. Epub 2012/05/29.
26. Bitetto D, Fabris C, Fornasiere E, Pipan C, Fumolo E, Cussigh A, et al. Vitamin D supplementation improves response to antiviral treatment for recurrent hepatitis C. Transplant international : official journal of the European Society for Organ Transplantation. 2011;24(1):43-50. Epub 2010/07/24.

27. Beinhardt S, Aberle JH, Strasser M, Dulic-Lakovic E, Maieron A, Kreil A, et al. Serum level of IP-10 increases predictive value of IL28B polymorphisms for spontaneous clearance of acute HCV infection. Gastroenterology. 2012;142(1):78-85 e2. Epub 2011/12/24.
28. Marek B, Kajdaniuk D, Mazurek U, Janczewska-Kazek E, Kos-Kudla B, Strzalka B, et al. TGF-beta1 mRNA expression in liver biopsy specimens and TGF-beta1 serum levels in patients with chronic hepatitis C before and after antiviral therapy. Journal of clinical pharmacy and therapeutics. 2005;30(3):271-7.

Information for participant

Information for participant

Title of research: Correction of vitamin D deficiency in chronic hepatitis C patients who had sustained virologic response after direct-acting agent therapy: Effect on serum hepatic fibrogenesis markers

Principle investigators:

1. Dr. Piyawat Komolmit
2. Dr. Supachaya Sriphoosanaphan

Affiliation: Division of Gastroenterology and Hepatology
Department of Medicine, Faculty of Medicine
Chulalongkorn University
Tel. 02-2564000 ext. 4356 no. 2

To participants

You are invited to participate in this clinical trial as you have chronic hepatitis C and found to have vitamin D deficiency. Before your agreement to involve in this trial, please read this document thoroughly for your understanding of the reason of the research and the detail of the protocol. If you have any questions, please ask our investigator team or principle investigators.

You can ask for advice from your family, friend or your general practitioner. You have time for your decision freely. If you decide to participate in this trial, please sign the agreement at the end of this document.

Background

Chronic hepatitis C is a common cause of chronic liver disease which resulting in progressive cirrhosis and hepatocellular carcinoma. Mechanisms behind hepatic fibrogenesis are active infection and inflammation which induce hepatic stellate cells (HSCs) to the activated myofibroblast like cells called activated HSCs. Fibrogenic cytokines responsible for these initial processes are transforming growth factor (TGF β_1), platelet derived growth factor (PDGF) and connective tissue growth factor. Activated HSCs proliferate and secrete more and more extracellular matrix (ECM) components and ultimately the liver reaches the state of cirrhosis.

Vitamin D (VD) is previously believed to be solely involved in calcium homeostasis. Nowadays, it has been known that VD acts as a hormone, autocrine and paracrine, involves in various

kinds of body regulations. It involves in both innate and adaptive immunes responses and keeps balance of the T and B cells ‘cytokines.

Degree of VD deficiency is worsening in CHC patients with progressive liver cirrhosis. Several studies demonstrated that chronic hepatitis C (CHC) patients with VD deficiency had lower sustained virological response (SVR) than the patients with normal VD levels when treated by interferon/ribavirin regimens. In addition, VD supplement help to reduce inflammation and improve SVR. Recent data suggest that VD inhibits HSCs proliferation and hepatic fibrogenesis.

Our previous study demonstrated that restoration of VD deficiency in patients with CHC improves the serum fibrogenesis markers. There is a change toward fibrolytic activities. This evidence highlights the role of VD in human hepatic fibrogenesis.

The possible explanations of protective role of VD in liver fibrosis could be due to three main mechanisms.

4. HCV viral replication
5. Inflammation reduction
6. Direct effect on hepatic fibrogenesis

However, little is still known about the exact properties of VD on hepatic fibrogenesis. In this study, we aim to explore and clarify the exact role of VD. We study the patients with CHC who underwent curative direct-acting antivirals (DAA) treatment. We believe that these patients do not have the viral replication and hepatic inflammation left. Therefore, we hypothesized that restoration of VD deficiency after curative treatment would further attenuate liver fibrosis, as assessed by the improvement of fibrogenesis markers; TGF-β_1, TIMPs, MMPs, and P3NP.

Objective of the study

1. To study the effect of 6-week supplementation of VD on the changes of fibrogenic cytokine, TGF β_1 as compared to placebo

2. To study the effect of 6-week supplement of VD on the changes of fibrolytic enzymes, TIMP-1, MMP-9, and P3NP as compared to placebo

Number of participants:

80 persons for screening and 60-75 patients will be enrolled into the study

Methods involved in this trial

After your agreement to participate in this trial, our investor team would like to check your blood (10 mL) for vitamin D and keep for measurement of various substances.

If you have vitamin D deficiency and have no conditions that should not be in the trial, we will arrange appointment to see the doctor for general physical examination and evaluation for the result of blood tests and receive the vitamin D or placebo tablets. The time period of the clinical trial is 6 weeks and you have to see the doctor twice (at baseline and at 6 weeks).

Responsibility of the participants

For success of this study, we would like to ask participants to have discipline to comply with the protocol. If you have any abnormal symptoms during this study, please contact the investigator team.

For your safety, you should not receive any vaccination or other medications by other doctors or from pharmacy. Please consult our investigator team if you need any question regarding other medications as they might have effect on vitamin D during the period of study.

Risk of the study

Any medications or event vitamins could have side effects of any severity. We would like to explain the risk and symptoms that might relate to the drugs involve in this study.

The chance of getting toxicity from vitamin is very low and the symptoms have been reported as tiredness, headache, anorexia, dry mouth, nausea and vomiting. The dosage of vitamin D in this trial is as of recommendation and the risk of adverse symptoms is low. However, if you experience any symptoms, please contact us for advice.

Risk from drawing blood sample

You may experience of pain at the puncture site, minor bleeding, ecchymosis, edema, syncope and local infection, which rarely happen.

Risk from other things

You might experience of some other symptoms not mention in this document as not seen before. For your safety, please report of any symptoms you may concern to the investigator team at any time.

If there are any new reports of any safety concern regarding the medications used in this trial, we will inform all participants as soon as possible and you may decide to continue or pull out from the study.

How to see the doctor for your concern of any adverse events

You can contact the principle investigators or the team at any time in case you experience of some symptoms or concern of any adverse events. Immediate advice or treatment will be provided.

Benefit from the study

To participate in this trial, your health might be improved and reduced in severity. However, this will not be a guarantee.

Other methods or managements for the participants

You have no need to be in this clinical trial for expecting of the treatment. As there might be other ways of treatment for your disease. You may ask the doctor or your GP before making decision to participate in this clinical trial.

Practical points for participants during the trial

Please read carefully

- Please give your information regarding your health and history of diseases or treatment.
- Please inform our team if you experience any symptoms of concern
- Please abstain from other medications, herbs or un prescribed drugs from pharmacy
- Please inform the investigator team in cases you receive other new medications during the study period
- Please bring and return the tablets that have left after finishing the trial

Adverse events or complications happened during the trial and responsibility

If you have any complications during the trial, you will receive immediate treatment. Our investigator team will responsible for the cost of treatment and you signature at the end of the document does not mean that you disclaim from your regular health scheme.

If you experience any adverse events, you could contact the principle investigators by phone any time.

Participation or withdrawn from clinical trial

To participate in this clinical trial is you right to make decision and you could withdraw from the trial at any time. Your decision will not have any risk or consequence to your regular treatment of your diseases.

Our investigator team will withdraw you from the trial for your safety or for other following reasons:

- You could not comply with our protocol.
- You receive other medications preclude in this study.
- You have pregnancy during the trial.
- You experience some adverse events or abnormal laboratory results that may risk for your health.
- You have moderate or severe allergic reaction to the study drugs.
- You receive other medication that preclude for the study protocol.

Measurement for protection of participants' data

Your data and your name will be protected from any publicity. In case of publication, the name or address of the participant will be protected and the participant code number will be used instead.

After your agreement, the investigators will have the right to exam your data even after the trial finished. If you could withdraw that right at any time by contacting and inform in person or in writing and send it to the principle investigator (address shown).

If you withdraw from the trial, your add on personal data will not be done. However, some data will be used for evaluation. You could not return to the study protocol again after withdrawal.

After your agreement, the investigator could inform your GP regarding the agreement for participation in the trial.

Right of the participants

As you decide to be in this trial, you have the right as following

- You will receive information of the trial
- The investigators will inform regarding method of the study, drugs and other tests.
- You will receive information of risk or adverse event from the medications
- You will receive information of the benefit of the trial
- You will receive information regarding other alternative treatment that might benefit to your disease.
- You will receive information of management of adverse events or complications.
- You could ask for more information regarding process of the study.
- You will receive information regarding how and when to withdraw from the study which could be any time.
- You will receive the consent form with the signatures and date
- You have the right to make decision whether or not to participate the trial without any influence or pressure from anyone.

If you do receive any compensation for your adverse events related to trial medication or you do not receive proper management as the explanation in this document, you could contact the principle investigators directly or report to Institutional Review Board, Faculty of Medicine, Chulalongkorn University at the office on the 3rd floor Mahidol Building, King Chulalongkorn Memorial Hospital, Rama 4 Road, Pathumwan, Bangkok 10330, Tel. 02-256-4455, ext. 14 or 15 during office hour.

Thank you for your cooperation

Consent form for agreement to participate in the trial

Consent form for agreement to participate in the trial

Title of research: Correction of vitamin D deficiency in chronic hepatitis C patients who had sustained virologic response after direct-acting agent therapy: Effect on serum hepatic fibrogenesis markers

Date of agreement: Date………….. Month………………Year………………

I, Mr./Mrs./MS…………………………………………………Age……..…….years
Current address……………………………………………………………………………..
Tel. ……………………………………… have read the information for the participant and agree to participate in this clinical trial.

I have received the copy of the consent form for participation in the trial and sign with the name and date include receiving the detail document for participant. I have received explanation regarding objective, period of study, methodology, risks that might happen and benefit of this trial. I have enough time to read and ask for any concern regarding the clinical trial and the investigators give all information without any hidden agenda.

I have the right to withdraw from the clinical trial at any time and without the need to explain the reasons. In addition, the withdrawal will not have any consequence to my disease management or my right to receive proper management.

The investigators confirm to protect the secrecy of my data and will reveal only on my permission.

Any investigation or examination of the data by other party including Institutional Review Board member have the right only to examine for the accuracy of the data. By this agreement, I accept for the examination of my previous health history.

The investigator agree to the participant that, in case of withdrawal, no more additional data will be kept and the data related to the participant will be abolished and could not be traced back to the participant.

I understand that I have a right to exam or correct my personal data and have a right of others to use my personal data by informing the investigators.

I understand that the research data including the health history will not be opened or report by participant name. And the data will be used to process by data correction, computer analysis of the data and then report only for scientific and clinical purpose.

I accept to sign this consent form for participation in the clinical trial with approval.

…………………………………………………………Participant signature

…………………………………………………………Block letters

Date…………Month……………Year……………

I have explained the information regarding this clinical trial including objective, methodology, risk or benefit of the trial and the participant as the above name has signed for the agreement to comply with the trial.

…………………………………………………………Investigator signature

…………………………………………………………Block letters

Date………….Month……………Year………………

……………………………………………………… Witness signature

………………………………………………………Block letters

Date………….Month…………Year……………

Case record form

Case number.................................
Date ..../........../............ (DD/MM/YY)

Case Record Form

แบบฟอร์มบันทึกข้อมูลผู้ป่วยโครงการวิจัย

ผลของการแก้ไขภาวะขาดวิตามินดีในผู้ป่วยไวรัสตับอักเสบซีที่ตอบสนองต่อยาต้านไวรัสกับการเปลี่ยนแปลงของระดับไซโตไคน์และเอ็นไซม์ที่เกี่ยวเนื่องกับการเพิ่มพังผืดในตับ

Correction of Vitamin D Deficiency in Chronic Hepatitis C Patients who Had Sustained Virologic Response After Direct-acting Agent Therapy: Effect on Serum Hepatic Fibrogenesis Markers

Baseline Characteristics

Age .... years

Sex ☐ Male ☐ Female

Weight .... kg.

Height .... cm.

BMI kg/m2

Fibroscan .... kpa

MRE ....

Ultrasound ....

Underlying disease

☐ No ☐ Yes, specify ....
....

Current medications

☐ No ☐ Yes, specify ....
....

HCV diagnosis:

Duration of HCV diagnosis .... months

HCV risk factor(s)

- Heterosexual
- Homosexual
- IVDU
- Blood transfusion
- Tattoo
- Unknown
- Other please define ....

Date of start treatment (DD/MM/YYYY)

............../.............../.................

Date of end of treatment (DD/MM/YYYY)

............../.............../.................

Baseline 25(OH) Vitamin D level (ng/mL.)

□ < 10 ng/mL
□ 10 -20 ng/mL
□ 20-30 ng/mL
□ > 30 ng/mL

Serology profile

Anti HCV .... HBsAg ....

Anti-HBs .... Anti HIV ....

Baseline HCV genotype before treatment ....

Previous Tx Regimen	Start date	Stop date	VL	Note

Baseline Laboratory

Complete Blood Count

Hct .... Hb........... MCV ....

Wbc ....

% PMN .... % Lymph ....

% Mono .... % Eos. ....

Platelet Count ....

PT .... INR .... PTT ....

Liver Function Test

TB .... DB ....

Albumin .... Globulin ....

SGOT .... SGPT ....

ALP ....

Blood chemistry

BUN .... Cr ....

Na .... K ....

Cl .... CO2 ....

Ca .... Phosphate ....

Others ....

Vitamin D 25(OH)VD

Baseline ....ng/mL

After treatment .... ng/mL

TGF-β1 level

Baseline ....ng/mL

After treatment ....ng/mL

TIMP-1 level

Baseline ....ng/mL

After treatment ....ng/mL

MMP-9 level

Baseline ....ng/mL

After treatment ....ng/mL

P3NP level

Baseline ....ng/mL

After treatment ....ng/mL

Laboratory Follow up

Date (DD/MM/YYYY) ....

Hct .... Hb........... MCV .... Wbc .... % PMN .... % Lymph ....

ANC .... Platelet Count .... PT .... INR .... PTT ....

BUN .... Cr .... TB .... DB .... Albumin .... Globulin ....

SGOT .... SGPT .... ALP ....

Ca .... Phosphate ....

Record by ....

Date ....
