# Supplementary material for: Effect of vitamin D supplementation in patients with chronic hepatitis C after direct-acting antiviral treatment: a randomized, double-blind, placebo-controlled trial
Source: PeerJ. 2021 Feb 9;9:e10709. doi: 10.7717/peerj.10709 (PMC7879942; doi:10.7717/peerj.10709)
Supplement: Supplemental Information 5 [file peerj-09-10709-s005.pdf]

Related Manuscript (Thai)

Effect of vitamin D supplementation in patients with chronic hepatitis C after direct-acting antiviral treatment: a randomized, double-blind, placebo-controlled trial

Supachaya Sriphoosanaphan[1,2], Kessarin Thanapirom[1,2,3], Sirinporn Suksawatamnuay[1,2,3], Panarat Thaimai[1], Sukanya Sittisomwong[1], Kanokwan Sonsiri[1], Nunthiya Srisoonthorn[2], Nicha Teeratorn[1], Nattaporn Tanpowpong[4], Bundit Chaopathomkul[4], Sombat Treeprasertsuk[1], Yong Poovorawan[5], Piyawat Komolmit*[1,2,3]

1. Division of Gastroenterology, Department of Medicine, Faculty of Medicine, Chulalongkorn University and King Chulalongkorn Memorial Hospital, Bangkok, Thailand
2. Centre of Excellence in Liver Diseases, King Chulalongkorn Memorial Hospital, Bangkok, Thailand Thai Red Cross, Pathumwan, Bangkok Thailand
3. Liver Fibrosis and Cirrhosis Research Unit, Chulalongkorn University, Bangkok, Thailand
4. Department of Radiology, Faculty of Medicine Chulalongkorn University, King Chulalongkorn Memorial Hospital, Bangkok, Thailand
5. Centre of Excellence in Clinical Virology, Faculty of Medicine, Chulalongkorn University, Bangkok, Thailand

*Corresponding author: Piyawat Komolmit, MD, PhD.

Division of Gastroenterology, Department of Medicine, Faculty of Medicine, Chulalongkorn University, Bangkok 10330, Thailand Tel: +662-2564265, +669-47825195
Email: pkomolmit@yahoo.co.uk

Table of contents **Page**

1. Research Protocol in Thai 2
2. Supplement documents
    a. Information for participant 14
    b. Informed consent sheet 22
    c. Case record form 25

Research Protocol

1. **ชื่อโครงการ** (Proposal Title)

(ภาษาไทย) ผลของการแก้ไขภาวะขาดวิตามินดีในผู้ป่วยไวรัสตับอักเสบซีที่ตอบสนองต่อยาต้านไวรัสกับการเปลี่ยนแปลงของระดับไซโตไคน์และเอ็นไซม์ที่เกี่ยวเนื่องกับการเพิ่มพังผืดในตับ

(ภาษาอังกฤษ) Correction of Vitamin D Deficiency in Chronic Hepatitis C Patients who Had Sustained Virologic Response After Direct-acting Agent Therapy: Effect on Serum Hepatic Fibrogenesis Markers

2. **ชื่อคณะผู้วิจัย** (Investigators)

ผู้วิจัยหลัก ผู้ช่วยศาสตราจารย์นายแพทย์ปิยะวัฒน์ โกมลมิศร์

ผู้วิจัยร่วม นายแพทย์ศุภพัชญ ศรีภูษณาพรรณ

สังกัด หน่วยโรคระบบทางเดินอาหารและตับ ภาควิชาอายุรศาสตร์ คณะแพทยศาสตร์ โรงพยาบาลจุฬาลงกรณ์ ตึกภูมิสิริมังคลานุสรณ์ เลขที่ 1873 ถนน พระรามที่ 4 แขวงปทุมวัน เขตปทุมวัน กรุงเทพมหานคร 10330 โทร. 02-256-4265

3. **ความสำคัญ และที่มาของปัญหาการวิจัย** (Background and rationale)

การติดเชื้อไวรัสตับอักเสบซีเป็นสาเหตุที่สำคัญชนิดหนึ่งที่ทำให้เกิดภาวะโรคตับเรื้อรัง นำไปสู่การเกิดภาวะตับแข็งและมะเร็งตับได้ในที่สุด[1, 2] ซึ่งเมื่อการติดเชื้อไวรัสตับอักเสบซีดำเนินสู่ภาวะเรื้อรังก็จะกระตุ้นการเกิดพังผืดในตับเพิ่มมากขึ้น กลไกประการหนึ่งเกิดจากเซลล์ในระบบภูมิคุ้มกันมีการสร้างและหลั่งไซโตไคน์หลายชนิดไปกระตุ้นการทำงานของ hepatic stellate cells (HSCs) ซึ่งเป็นเซลล์หลักในการสร้างพังผืดภายในตับ โดยเมื่อ HSCs ถูกกระตุ้น จะทำให้มีการหลั่งไซโตไคน์และเอ็นไซม์ที่เกี่ยวข้องกับการสร้างพังผืดที่เพิ่มขึ้น เช่น transforming growth factor (TGF-β) , tissue inhibitor of matrix metalloproteinases (TIMP) และมีการลดลงของเอ็นไซม์ที่ยับยั้งการสร้างพังผืด ได้แก่ matrix metalloproteinases (MMPs) ทำให้เกิดกระบวนการสร้างพังผืดอย่างต่อเนื่องและมีการสะสมของ extracellular matrix proteins ในปริมาณที่มากขึ้นภายในตับ[3] ถ้ากระบวนการดังกล่าวยังดำเนินต่อไปก็จะส่งผลให้เกิดภาวะตับแข็งได้ในที่สุด

วิตามินดี แต่เดิมนั้นมีความเข้าใจว่า เป็นสารที่มีหน้าที่สำคัญในการควบคุมการทำงานของกระดูกและระดับแคลเซียมในร่างกาย แต่ในปัจจุบันพบว่า นอกเหนือจากหน้าที่สำคัญดังกล่าวแล้ว วิตามินดียังมีผลต่อการควบคุมและการปรับเปลี่ยนการทำงานของระบบภูมิคุ้มกันทั้งชนิดไม่เฉพาะเจาะจง และ แบบเฉพาะเจาะจง (innate and adaptive immunity) ร่วมด้วย โดยกลไกของวิตามินดีที่มีต่อระบบภูมิคุ้มกันจะเกี่ยวข้องกับการควบคุมความสมดุลในการสร้างไซโตไคน์ของทีและบีเซลล์[4]

จากการศึกษาที่ผ่านมาพบว่า ระดับวิตามินดีที่ลดลงในผู้ป่วยไวรัสตับอักเสบซีชนิดเรื้อรังสัมพันธ์กับการทำงานของตับที่ลดลงและมีความรุนแรงของโรคที่มากขึ้น[5-7] การได้รับวิตามินดีสามารถลดการสร้างไซโตไคน์ที่เกี่ยวข้องกับกระบวนการ

อักเสบลงได้ในผู้ป่วยไวรัสตับอักเสบซี และการได้รับวิตามินดีเสริมร่วมกับการรักษามาตรฐานในผู้ป่วยไวรัสตับอักเสบซีพบว่าสามารถเพิ่มโอกาสการหายของโรค [sustained virological response (SVR)] ได้อย่างชัดเจน[8, 9] และจากการศึกษาล่าสุดพบว่าการให้วิตามินดีมีผลลดการแบ่งตัวของ HSCs และลดการสร้างพังผืดภายในตับจากการศึกษาในห้องปฏิบัติการ[10, 11]

นอกจากนี้จากการศึกษาเบื้องต้นทางกลุ่มวิจัยพบว่าการแก้ไขภาวะขาดวิตามินดีในผู้ป่วยไวรัสตับอักเสบซีสามารถลดระดับของ TGF-β_1 และ TIMP-1 ได้ รวมทั้งมีการเพิ่มระดับของเอ็นไซม์ MMP-2 และ MMP-9 อย่างมีนัยสำคัญทางสถิติ ซึ่งบ่งชี้ว่าการแก้ไขภาวะขาดวิตามินดีมีผลช่วยลดกระบวนการสร้างพังผืดในตับ[12] นับว่าเป็นหลักฐานแรกที่แสดงให้เห็นบทบาทของวิตามินดีในการลดพังผืดในตับในมนุษย์

หลักฐานในปัจจุบันเชื่อว่าวิตามินดีสามารถลดกระบวนการสร้างพังผืดในตับในผู้ป่วยไวรัสตับอักเสบซีได้โดยอาศัย 3 กลไกหลักร่วมกัน ได้แก่

1. ยับยั้งการแบ่งตัวของไวรัสตับอักเสบซี
2. ลดภาวะการอักเสบภายในเนื้อตับ
3. ลดกระบวนการสร้างพังผืดในตับโดยตรง

อย่างไรก็ตามปัจจุบันยังไม่มีข้อมูลหรือการศึกษาใดที่สามารถอธิบายกลไก ความสัมพันธ์และบทบาทของวิตามินดีต่อการสร้างพังผืดในตับได้อย่างชัดเจน

ในผู้ป่วยไวรัสตับอักเสบซีที่หายขาดจากการรักษาด้วยยาต้านไวรัส [Direct acting agent (DAA)] ผู้ป่วยกลุ่มนี้จะมีลดลงของการเกิดพังผืดในตับ นอกจากนี้กระบวนการสร้างพังผืดในตับจะไม่ถูกรบกวนจากการแบ่งตัวของไวรัสตับอักเสบซีและการอักเสบของเนื้อตับอีกต่อไป ผู้วิจัยจึงตั้งสมมุติฐานว่าหากเราทำการศึกษาการแก้ไขภาวะขาดวิตามินดีในผู้ป่วยกลุ่มนี้น่าจะยังสามารถช่วยลดกระบวนการสร้างพังผืดและช่วยส่งเสริมทำให้ภาวะพังผืดในตับลดลงได้ ดังนั้นการแก้ไขภาวะขาดวิตามินดีในผู้ป่วยไวรัสตับอักเสบซีที่หายขาดจากการรักษาด้วย DAA จะทำให้เกิดการเปลี่ยนแปลงของระดับ TGF-β_1, TIMPs MMPs และ P3NP.

4. **ทบทวนวรรณกรรมที่เกี่ยวข้อง** (Review of related literatures)

การติดเชื้อไวรัสตับอักเสบซีเป็นสาเหตุที่สำคัญชนิดหนึ่งที่ทำให้เกิดภาวะโรคตับอักเสบเรื้อรัง นำไปสู่การเกิดภาวะตับแข็งและมะเร็งตับ ในระยะแรกของการติดเชื้อไวรัสตับอักเสบซีชนิดเฉียบพลับ ระบบภูมิคุ้มกันชนิดโดยกำเนิด (innate immune responses) จะเข้ามาทำหน้าที่ในการกำจัดเชื้อ แต่เมื่อใดก็ตามที่ร่างกายไม่สามารถกำจัดเชื้อได้สำเร็จในระยะเฉียบพลันจะทำให้ร่างกายจะมีการตอบสนองโดยการปรับระบบภูมิคุ้มกันซึ่งเกี่ยวข้องกับการกระตุ้นบีและทีเซลล์ (adaptive immune responses: B and T cells) เกิดภาวะตับอักเสบเรื้อรังตามมา ในระยะการติดเชื้อไวรัสตับอักเสบซีเรื้อรังการตอบสนองต่อระบบภูมิคุ้มกันดังกล่าวจะมีการสร้างและการหลั่งไซโตไคน์ (cytokines) โดยเฉพาะทีเซลล์ลิมโฟซัยท์ ชนิดซีดี 8 (CD 8, cytotoxic T cells) มีผลให้เกิดการกระตุ้นขบวนการการอักเสบอย่างต่อเนื่องส่งผลโดยตรงต่อตับทำให้เกิดการทำลายตับ[13] และเกิดตับอักเสบเรื้อรังได้ในที่สุด

การเกิดพังผืดในตับ (liver fibrosis) เกิดจากมีการสะสมของ extracellular matrix proteins ในปริมาณที่มากขึ้น เป็นผลจากความไม่สมดุลกันระหว่างการสร้างพังผืดที่เพิ่มขึ้นกับการย่อยสลายพังผืดที่ลดลงภายในตับ โดยภาวะใดๆก็ตามที่ทำให้เกิดการอักเสบภายในตับแบบเรื้อรังและต่อเนื่อง เช่น การดื่มสุรา การได้รับยาหรือสารพิษที่มีผลต่อตับ การติดเชื้อไวรัสตับ

อักเสบชนิดบีหรือซีชนิดเรื้อรัง ปัจจัยดังกล่าวข้างต้นทำให้มีการกระตุ้นระบบภูมิคุ้มกันชนิดทีเซลล์ลิมโฟซัยท์ ให้มีการสร้างและหลั่งไซโตไคน์หลายชนิดไปกระตุ้นการทำงานของ hepatic stellate cells (HSCs)[3, 14] ซึ่งเป็นเซลล์เริ่มต้นที่ทำให้มีการกระตุ้นการสร้างพังผืดภายในตับที่มากขึ้น โดยเมื่อ HSCs ถูกกระตุ้น จะทำให้มีการหลั่งไซโตไคน์ที่เกี่ยวข้องกับการสร้างพังผืดที่เพิ่มขึ้น (fibrogenic cytokines) เช่น TGF-β , platelet derived growth factor (PDGF) , connective tissue growth factor (CTGF) ซึ่งตัวที่สำคัญคือ TGF-β_1

TGF-β เป็นไซโตไคน์ที่ถูกสร้างขึ้นได้จากหลายภาวะและหลายกลุ่มโรค[15] โดยการศึกษาที่เกี่ยวข้องกับโรคตับพบว่า TGF-β สัมพันธ์กับการสร้างและการสะสมของพังผืดและคอลลาเจน (collagen) ภายในตับและถูกสร้างโดย HSCs เป็นส่วนใหญ่ โดยมีการศึกษาในสัตว์ทดลองที่ใช้สารเคมี (CCl_4) กระตุ้นให้เกิดพังผืดในตับพบว่าการสะสมของคอลลาเจนที่เพิ่มขึ้นภายในตับสัมพันธ์กับการตรวจพบ TGF-β ในเนื้อเยื่อตับ (TGF-β expression)[16] และระดับ TGF-β_1 ในเนื้อเยื่อตับของผู้ป่วยไวรัสตับอักเสบซีชนิดเรื้อรังก็มีความสัมพันธ์กับระดับ TGF-β_1 ในเลือดโดยมีค่าความสัมพันธ์ (r) เท่ากับ 0.83 นอกจากนี้ระดับของ TGF-β_1 ในผู้ป่วยไวรัสตับอักเสบซีชนิดเรื้อรังยังสัมพันธ์กับระดับความรุนแรงของพังผืดในตับ(degree of fibrosis) อย่างมีนัยสำคัญทางสถิติ ($p < 0.01$)[16, 17] และมีค่าความสัมพันธ์(r) เท่ากับ 0.495[18] มีการศึกษาเปรียบเทียบระดับ TGF-β_1 ก่อนและหลังการได้รับการรักษาด้วย Interferon-α พบว่าระดับ TGF-β_1 ลดลงอย่างมีนัยสำคัญทางสถิติหลังได้รับการรักษาด้วยยาดังกล่าวและยังพบว่าระดับของพังผืดในตับก็ลดลงอย่างมีนัยสำคัญทางสถิติด้วยเช่นเดียวกัน[17] จากข้อมูลที่ผ่านมาในปัจจุบันจึงได้เริ่มมีการนำตัววัดพังผืดของตับในซีรั่ม โดยเฉพาะ TGF-β_1 มาใช้ช่วยในการวินิจฉัยระยะของพังผืดตับ (Stage of fibrosis) เพิ่มมากขึ้น[19, 20] และระดับที่มากกว่า 115 ng/ml จะมีความเสี่ยงสูงต่ออัตราการเกิดพังผืดตับที่เพิ่มขึ้นอย่างรวดเร็ว (rapid progression)[16]

วิตามินดีมีหน้าที่สำคัญในการควบคุมการทำงานของแคลเซียมและกระดูก แต่ในปัจจุบันมีข้อมูลของวิตามินดีที่เพิ่มมากขึ้นนอกเหนือจากการควบคุมการทำงานของโครงร่างและกระดูก เช่น การควบคุมและการปรับเปลี่ยนการทำงานของระบบภูมิคุ้มกันทั้งชนิด innate และ adaptive (immunomodulation) โดยกลไกของวิตามินดีที่มีต่อระบบภูมิคุ้มกันจะเกี่ยวข้องกับการควบคุมสมดุลในการสร้างไซโตไคน์ของทีและบีเซลล์ โดยพบว่าวิตามินดีจะยับยั้งการทำงานของ antigen presenting cells จากการยับยั้งการหลั่ง interleukine 2 (IL2) ส่งผลให้มีการเพิ่มจำนวนของ T helper 2 มากขึ้นและลดจำนวนของ T helper 1 ลง นอกจากนี้วิตามินดียังมีผลต่อการยับยั้งการหลั่งไซโตไคน์จาก T helper 1 (IL 2, IFN-γ) และกระตุ้นการหลั่งไซโตไคน์จาก T helper 2 มากขึ้น การที่วิตามินดีสามารถยับยั้งการสร้างและลดการหลั่งไซโตไคน์จาก T helper 1 ลงได้จะทำให้ลดการสร้าง cytotoxic T cells และช่วยปกป้องเซลล์หรืออวัยวะเป้าหมายไม่ให้ถูกทำลายลงได้[4]

การขาดวิตามินดีในผู้ป่วยที่เป็นโรคตับเรื้อรังส่วนหนึ่งอธิบายได้จากการที่ตับสูญเสียความสามารถในการสังเคราะห์วิตามินดีจากการทำงานของตับที่ลดลง แต่ในบางการศึกษากลับพบว่าระดับของวิตามินดีในผู้ป่วยโรคตับเรื้อรังระยะสุดท้ายยังอยู่ในเกณฑ์ปกติ[21] ดังนั้นจึงเชื่อว่ายังมีปัจจัยอื่นๆที่อาจมีผลต่อการขาดวิตามินดีได้เช่นกัน เช่น การได้รับแสงแดดที่ไม่เพียงพอ การขาดสารอาหาร (malnutrition) หรืออาจจะเป็นผลของไวรัสตับอักเสบซีต่อการทำงานของวิตามินดี[22]

ผู้ป่วยไวรัสตับอักเสบซีพบอุบัติการณ์การขาดวิตามินดีที่รุนแรง [25(OH)D3 < 10 ng/mL] เพิ่มสูงขึ้นทั้งในผู้ป่วยที่มีระดับพังผืดในตับที่รุนแรง (Metavir score $\geq$ F2)และในผู้ป่วยที่ไม่มีพังผืดในตับหรือมีในระดับที่น้อยมาก (Metavir score F0-F1) เมื่อเปรียบเทียบกับคนปกติ (healthy subjects) อย่างมีนัยสำคัญทางสถิติ[22] ในผู้ป่วยไวรัสตับอักเสบซีเรื้อรังที่มีระดับพังผืดอยู่เพียงแค่ในระยะเริ่มต้น (F0-F2) และมีระดับวิตามินดีที่ลดลง ยังสัมพันธ์กับอัตราการเกิดพังผืดตับที่เร็วเพิ่มขึ้น (rapid

fibrosis progression)เมื่อเปรียบเทียบกับผู้ป่วยที่มีระดับวิตามินดีที่ปกติจะมีอัตราการเกิดพังผืดตับที่ช้ากว่า[23] ผู้ป่วยที่มีการขาดวิตามินดีที่รุนแรงมีอัตราการหายจากโรคไวรัสตับอักเสบซีชนิดเรื้อรัง (SVR) น้อยกว่าผู้ป่วยที่มีการขาดวิตามินดีที่ไม่รุนแรง (ร้อยละ 50 และ 81 ตามลำดับ) เช่นเดียวกัน[22] ระดับของวิตามินดีที่ลดลงในผู้ป่วยไวรัสตับอักเสบซีเรื้อรังชนิดที่ 1 สัมพันธ์กับระยะของการเกิดพังผืดในตับที่สูงขึ้น (stage of fibrosis) และยังสัมพันธ์กับการเพิ่มขึ้นของการอักเสบภายในตับอย่างมีนัยสำคัญทางสถิติ[7, 8]

และจากการศึกษาที่ผ่านมาผลของการได้รับวิตามินดีเสริมต่อไวรัสตับอักเสบซีชนิดเรื้อรังพบว่า วิตามินดีสามารถยับยั้งการแบ่งตัวของไวรัสตับอักเสบซีได้ในห้องทดลอง (*in vitro*)[25] การได้รับวิตามินดีเสริมร่วมกับการรักษามาตรฐานในผู้ป่วยไวรัสตับอักเสบซีสามารถเพิ่มอัตราการหายจากโรค (SVR) ได้[9, 26] การให้วิตามินดีมีผลลดการแบ่งตัวของ HSCs และลดการสร้างพังผืดภายในเนื้อเยื่อตับในห้องปฏิบัติการทดลอง[10, 11] โดยกลไกเชื่อว่าการได้รับวิตามินดีเสริมสามารถลดการสร้างไซโตไคน์ที่เกี่ยวข้องกับขบวนการอักเสบลงได้ในผู้ป่วยไวรัสตับอักเสบซี[27]

นอกจากนี้จากการศึกษาเบื้องต้นทางกลุ่มวิจัยพบว่าการแก้ไขภาวะขาดวิตามินดีในผู้ป่วยไวรัสตับอักเสบซีสามารถลดระดับของ TGF-β_1 และ TIMP-1 รวมทั้งมีการเพิ่มระดับของเอ็นไซม์ MMP-2 และ MMP-9 อย่างมีนัยสำคัญทางสถิติ ซึ่งบ่งชี้ว่าการแก้ไขภาวะขาดวิตามินดีมีผลช่วยลดกระบวนการสร้างพังผืดในตับ[12]

จากข้อมูลที่กล่าวมาข้างต้นเชื่อว่าวิตามินดีสามารถลดกระบวนการสร้างพังผืดในตับในผู้ป่วยไวรัสตับอักเสบซีได้โดยอาศัย 3 กลไกหลักร่วมกัน ได้แก่ การยับยั้งการแบ่งตัวของไวรัสตับอักเสบซี, การลดภาวะการอักเสบภายในเนื้อตับและการลดกระบวนการสร้างพังผืดในตับโดยตรง อย่างไรก็ตามปัจจุบันยังไม่มีหลักฐานหรือการศึกษาใดที่สามารถอธิบายกลไกและบทบาทของวิตามินดีต่อการสร้างพังผืดในตับได้อย่างชัดเจน

ดังนั้น จึงเป็นที่น่าสนใจเป็นอย่างยิ่งว่า หากเราทำการศึกษาในกลุ่มผู้ป่วยไวรัสตับอักเสบซีที่หายขาดจากการรักษาด้วยยาต้านไวรัส นั่นคือกระบวนการสร้างพังผืดในตับจะไม่ถูกกระทบจากการแบ่งตัวของไวรัสตับอักเสบซีและการอักเสบของเนื้อตับ การแก้ไขภาวะขาดวิตามินดีจะยังมีผลช่วยลดกระบวนการสร้างพังผืดได้หรือไม่ จึงเป็นที่มาของการศึกษางานวิจัยนี้ที่ต้องการทราบถึงผลของการแก้ไขภาวะขาดวิตามินดีเสริมในผู้ป่วยไวรัสตับอักเสบซีที่หายขาดจากการักษาต่อการเปลี่ยนแปลงของระดับตัววัดพังผืดของตับในซีรั่ม

5. วัตถุประสงค์ของการวิจัย (Objective)

<u>วัตถุประสงค์(หลัก)</u> เพื่อประเมินผลของการได้รับวิตามินดีเสริมในผู้ป่วยไวรัสตับอักเสบซีที่หายขาดจากการรักษาด้วย DAA ที่มีภาวะขาดวิตามินดีต่อการเปลี่ยนแปลงของระดับ TGF-β_1 เมื่อรับประทานต่อเนื่อง 6 สัปดาห์เปรียบเทียบกับกลุ่มควบคุม

<u>วัตถุประสงค์(รอง)</u> เพื่อประเมินผลของการได้รับวิตามินดีเสริมในผู้ป่วยไวรัสตับอักเสบซีที่หายขาดจากการรักษาด้วย DAA ที่มีภาวะขาดวิตามินดีต่อการเปลี่ยนแปลงของระดับ TIMP-1, MMP-9 และ P3NP เมื่อรับประทานต่อเนื่อง 6 สัปดาห์เปรียบเทียบกับกลุ่มควบคุม

6. คำถามของการวิจัย/สมมติฐาน (Hypothesis)

<u>คำถามหลัก</u> การให้วิตามินดีเสริมในระยะ 6 สัปดาห์ แก่ผู้ป่วยไวรัสตับอักเสบซีที่หายขาดจากการรักษาด้วย DAA ที่มีภาวะขาดวิตามินดี จะมีผลต่อการเปลี่ยนแปลงของระดับไซโตไคน์และเอ็นไซม์ที่เกี่ยวเนื่องกับการเพิ่มพังผืดในตับในซีรั่มหรือไม่

7. **คำสำคัญ** (Key words)

- TGF-β_1
- Vitamin D Supplement
- Chronic Hepatitis C
- Liver fibrosis

8. **รูปแบบการวิจัย** (Research design)

การศึกษาแบบสุ่มและปกปิดสองทางเปรียบเทียบกับยาหลอก (Randomized double-blind placebo controlled trial)

9. **ระเบียบวิธีการวิจัย** (Research methodology)

ประชากรเป้าหมาย คือ ผู้ป่วยไวรัสตับอักเสบซีที่หายขาดจากการรักษาด้วย DAA และมีภาวะขาดวิตามินดีที่มารับการรักษาที่โรงพยาบาลจุฬาลงกรณ์

กฎเกณฑ์ในการคัดเลือกเข้ามาศึกษา (Inclusion criteria) คือ คนไข้ในคลินิกโรคระบบทางเดินอาหารและตับ โรงพยาบาลจุฬาลงกรณ์ ที่

1. ผู้ป่วยที่ได้รับการวินิจฉัยเป็นไวรัสตับอักเสบซีที่มี SVR ภายหลังการรักษาด้วย DAA ภายในระยะเวลา 1 ปี
2. อายุมากกว่า 18 ปี
3. ระดับ 25-OH vitamin D < 30 ng/ml
4. มีพังผืดในตับวัดโดยมีหลักฐานจาก

    4.1. Fibroscan โดยมีค่า transient elastogram > 7.1 kPa

    4.2. Magnetic resonance elastography (MRE)

    4.3. Ultrasound elastography

กฎเกณฑ์ในการคัดเลือกออกจากการศึกษา (Exclusion criteria) คือ

1. ผู้ป่วยติดเชื้อเอชไอวี ผู้ป่วยโรคออโตอิมมูน ผู้ป่วยที่ได้รับการวินิจฉัยว่าเป็นโรคหอบหืดเรื้อรังหรือโรคปอดอุดกั้นทางเดินหายใจ (Asthma or chronic obstructive pulmonary disease) และผู้ป่วยโรคมะเร็งทุกชนิด
2. ผู้ป่วยที่ได้รับการวินิจฉัยเป็นโรคตับอักเสบเรื้อรังหรือตับแข็งจากสาเหตุอื่น เช่น การติดเชื้อไวรัสตับอักเสบบี (chronic viral hepatitis B), autoimmune hepatitis, nonalcoholic fatty liver disease (วินิจฉัยจากอัลตราซาวด์), alcoholic liver disease (ปริมาณสุราที่ดื่มมากกว่า 60 กรัมต่อวันเป็นระยะเวลามากกว่าหรือเท่ากับ 10 ปี)
3. ผู้ป่วยที่กำลังตั้งครรภ์และ/หรือให้นมบุตร
4. ผู้ป่วยที่กำลังเจ็บป่วยติดเชื้อไวรัสหรือแบคทีเรีย (active viral or bacterial infection)
5. ผู้ป่วยที่เคยได้รับสเตียร์รอยด์หรือยากดภูมิคุ้มกันมาก่อนในช่วง 6 เดือนก่อนการคัดเลือกเข้าการศึกษา
6. ผู้ป่วยที่เคยได้รับการรักษาด้วย interferon มาก่อนในช่วง 12 เดือนก่อนการคัดเลือกเข้าการศึกษา

7. ผู้ป่วยที่ยังดื่มสุรามากกว่า 20 กรัมต่อวันในช่วง 6 เดือนที่ผ่านมาก่อนการคัดเลือกเข้าการศึกษา

8. ผู้ป่วยโรคไตวายเรื้อรังที่อัตราการกรองของไต (Glomerular Filtration Rate: GFR) น้อยกว่าหรือเท่ากับ 60 มิลลิลิตร ต่อนาที

9. ผู้ป่วยที่กำลังได้รับวิตามินดี

ขั้นตอนในการดำเนินการวิจัย

1. ชี้แจงวัตถุประสงค์ ขั้นตอนการวิจัย ประโยชน์ที่ผู้ป่วยจะได้รับ รวมถึงผลข้างเคียงที่อาจเกิดขึ้น โดยการอธิบายรายละเอียดกับผู้ป่วยทุกขั้นตอนตั้งแต่วัตถุประสงค์ ขั้นตอนการวิจัย ประโยชน์ที่ผู้ป่วยจะได้รับ รวมถึงผลข้างเคียงที่อาจเกิดขึ้นได้แก่ผู้ป่วยทุกรายด้วยตัวผู้วิจัยเองทุกคน และได้ขอความยินยอมจากผู้ป่วยเป็นลายลักษณ์อักษรทุกครั้งก่อนเริ่มทำงานวิจัย

2. ซักประวัติ ตรวจร่างกายตามแบบบันทึกข้อมูล และเก็บข้อมูลตามแบบสอบถาม

3. วัดระดับ serum 25-OH Vitamin D, TGF-β_1, TIMP-1, MMP-9 และ P3NP ที่เริ่มต้นก่อนการได้รับวิตามินดีหรือยาหลอก โดยอาสาสมัครที่จะเข้าเกณฑ์การวินิจฉัยภาวะขาดวิตามินดี คือต้องมีระดับ 25-OH vitamin D น้อยกว่า 30 นาโนกรัมต่อซีซี

4. แบ่งผู้ป่วยเป็นสองกลุ่ม โดยเทคนิค stratification block randomization กลุ่มหนึ่งได้รับวิตามินดีตามระดับ serum 25-OH vitamin D อีกกลุ่มได้รับยาหลอกซึ่งลักษณะทางกายภาพและรูปลักษณ์ของยาจะมีลักษณะเหมือนกันและมีส่วนผสมเหมือนกันยกเว้นตัวยาวิตามินดี เป็นระยะเวลา 6 สัปดาห์

5. วัดระดับ serum 25-OH Vitamin D, TGF-β_1, TIMP-1, MMP-9 และ P3NP ที่ 6 สัปดาห์หลังการได้รับวิตามินดีหรือยาหลอก

6. วัดผลโดยใช้แบบบันทึกข้อมูลและตรวจ TGF-β_1, TIMP-1, MMP-9 และ P3NP ด้วยวิธี quantitative analysis (ELISA), ระดับ 25-OH vitamin D และทำการวิเคราะห์ทางสถิติ

7. ผู้ป่วยในกลุ่มควบคุมทุกรายเมื่อเสร็จสิ้นงานวิจัยแล้วจะได้รับวิตามินดีเสริมทุกรายเพื่อให้ผู้ป่วยทุกรายที่ได้รับเข้าร่วมงานวิจัยได้ประโยชน์และไม่เสียโอกาสจากการได้รับวิตามินดีเสริม

8. ขนาดของวิตามินดีที่ผู้ป่วยในกลุ่มควบคุมทุกรายจะได้เสริมจะขึ้นกับระดับความรุนแรงที่ขาดของระดับ 25-OH vitamin D (ดังตารางที่ 1) โดยระยะเวลาที่จะได้รับทั้งสิ้นคือ 6 สัปดาห์ ส่วนในกลุ่มอาสาสมัครที่ได้รับการตรวจพบระดับ 25-OH vitamin D มากกว่า 30 นาโนกรัมต่อซีซี จะถือว่าไม่มีภาวะขาดวิตามินดีและไม่ได้อยู่ในเกณฑ์คัดเข้าการศึกษา ดังนั้นจึงไม่มีความจำเป็นในการให้วิตามินดีเสริม

Diagnosis	Vitamin D level (ng /mL)	Replacement Total dose (IU/week)	Ergocalciferol (D2) 20,000 IU/ tab	Duration
Optimal	>30	-	-	-
Mild deficiency (insufficiency)	20–30	60,000	2 tabs Monday and 1 tab Friday	6 weeks
Moderate deficiency	10–20	80,000	2 tabs Monday and 2 tab Friday	6 weeks
Severe deficiency	<10	100,000	3 tabs Monday and 2 tabs Friday	6 weeks

ตารางที่ 1 Protocol **สำหรับการให้วิตามินดีในระดับค่าวิตามินที่แตกต่างกัน**

การเก็บตัวอย่างเลือดเพื่อส่งตรวจ TGF-β_1, TIMP-1, MMP-9, P3NP

1. เก็บตัวอย่างเลือดใส่ clotted tube และตั้งทิ้งไว้ 30 นาทีที่อุณหภูมิห้อง
2. นำตัวอย่างเลือดมาปั่น 15 นาที
3. เก็บแยกซีรั่มไว้ที่อุณหภูมิ ≤ - 70 °c
4. ตรวจวัดระดับของ TGF-β_1, TIMP-1, MMP-9 และ P3NP ด้วยวิธี ELISA (R&D Systems) โดยวิธีที่ใช้ตรวจมีค่าสัมประสิทธิ์ของความแปรปรวนภายในชุดทดสอบ (Intra assay) ร้อยละ 1.9 - 2.9 และมีค่าสัมประสิทธิ์ของความแปรปรวนระหว่างชุดทดสอบ (Inter assay) ร้อยละ 6.4-9.3 และมีค่าเฉลี่ยความไว (ค่าเฉลี่ยที่น้อยที่สุดที่ตรวจได้) เท่ากับ 4.61 พิโคกรัมต่อมิลลิลิตร

การเก็บตัวอย่างเลือดเพื่อส่งตรวจระดับ 25-OH vitamin D

1. เก็บตัวอย่างเลือดใส่ clotted tube
2. ส่งตรวจทางห้องปฏิบัติการโรงพยาบาลจุฬาลงกรณ์ ด้วยวิธี Immunochemiluminescence

ขนาดตัวอย่าง (Sample size determination)

$$N/group = \frac{2\,(Z_{1-\alpha/2} + Z_{1-\beta})^2\,\sigma^2}{(X_1 - X_2)^2}$$

จากการศึกษาของ Komolmit P. และคณะ[(12)] กำหนดค่า α = 0.05, β = 0.05

จะได้ค่า N ต่อกลุ่ม โดยคำนวณอ้างอิงจาก serum hepatic fibrogenesis markers ได้แก่ TGF-β_1

N ทั้งหมด = 29 คน ในแต่ละกลุ่ม

กำหนด drop out อัตราร้อยละ 10 ดังนั้นจึงเพิ่มตัวอย่างทั้งหมดเป็น N = ~ 32 คนต่อกลุ่ม

ดังนั้น N เป็นทั้งหมด เท่ากับ 64 คน

10. **การรวบรวมข้อมูล** (Data collection)

เก็บข้อมูลจากหน่วยโรคทางเดินอาหารและตับ โรงพยาบาลจุฬาลงกรณ์

ผู้เก็บข้อมูลคือ ผู้ดำเนินการวิจัย และผู้บันทึกข้อมูลคือ ผู้ดำเนินการวิจัย

11. **การวิเคราะห์ข้อมูล** (Data analysis and Statistics)

ข้อมูลเชิงปริมาณ วิธีการทางสถิติที่ใช้ทดสอบสมมุติฐานเปรียบเทียบข้อมูลเชิงปริมาณ 2 กลุ่มที่ไม่เป็นอิสระต่อกัน (ก่อนและหลังการได้รับวิตามินดี) ทดสอบโดย paired t-test หรือ Wilcoxon signed-rank test ตามลักษณะการกระจายของข้อมูลและข้อมูล 2 กลุ่มที่เป็นอิสระต่อกัน (กลุ่มที่ได้รับวิตามินดีกับกลุ่มควบคุม) ทดสอบโดย unpaired t-test หรือ Mann-Whitney U-test ตามลักษณะการกระจายของข้อมูล

ข้อมูลเชิงคุณภาพ วิธีการทางสถิติที่ใช้วิเคราะห์โดย Chi-square test หรือ Fisher's exact test ตามความเหมาะสม

12. **ข้อพิจารณาทางจริยธรรม** (Ethical consideration)

หลักความเคารพในบุคคล (Respect for person) ผู้วิจัยจะเก็บความลับของอาสาสมัครโดยไม่มี identifier ในแบบบันทึกข้อมูลที่จะระบุถึงตัวอาสาสมัคร ผู้ที่จะสามารถเข้าถึงข้อมูลผู้ป่วยมีเฉพาะผู้วิจัยหลักเท่านั้น และอาสาสมัครทุกรายจะได้รับข้อมูลเกี่ยวกับรายละเอียดงานวิจัย วัตถุประสงค์ ประโยชน์และผลข้างเคียงที่เกิดขึ้นได้ อย่างครบถ้วนและได้รับความยินยอมจากผู้ป่วย

หลักการให้ประโยชน์ไม่ก่อให้เกิดอันตราย (Beneficial/Non-maleficence) การศึกษานี้มีประโยชน์กับอาสาสมัครในการให้วิตามินดีเสริมในผู้ที่ขาดวิตามินดีและอาจช่วยส่งเสริมการหายจากภาวะพังผืดในตับ ช่วยสนับสนุนและอธิบายกลไกการทำงานของวิตามินดีที่มีผลในการลดการเกิดขบวนการอักเสบและส่งผลให้เกิดการสร้างพังผืดในตับที่ลดลงได้ อาจได้รับความเสี่ยงจากการเจาะเลือดแต่จะแก้ไขด้วยการให้เจ้าหน้าที่ผู้ชำนาญในการเจาะเลือดเป็นผู้ดำเนินการเจาะเลือดให้

หลักความยุติธรรม (Justice) การศึกษานี้มีเกณฑ์การคัดเข้าและคัดออกอย่างชัดเจน และเป็นการศึกษาแบบสุ่มมีการกระจายความเสี่ยงและผลประโยชน์อย่างเท่าเทียมกันในอาสาสมัครทุกราย

13. **ผลหรือประโยชน์ที่คาดว่าจะได้รับจากงานวิจัย** (Expected benefit and application)

ทำให้ทราบถึงความสัมพันธ์ของการได้รับวิตามินดีว่าจะมีผลต่อการเปลี่ยนแปลงของระดับ TGF-β_1 , TIMP-1, MMP-9 และ P3NP หรือไม่ในผู้ป่วยไวรัสตับอักเสบซีที่หายขาดจาก DAA แล้ว เพื่อช่วยสนับสนุนและสามารถอธิบายกลไกการทำงานของวิตามินดีที่มีผลในการลดการเกิดขบวนการอักเสบและส่งผลให้เกิดการสร้างพังผืดในตับที่ลดลงได้ อีกทั้งยังส่งผลต่อการลดลงของพังผืดในตับหลังจากการรักษาจากไวรัสตับอักเสบซีเพิ่มมากขึ้น

14. **อุปสรรคที่อาจเกิดขึ้นและมาตรการแก้ไข** (Obstacles and strategies to solve the problems)

เนื่องจากงานวิจัยนี้ใช้ผู้ป่วยจำนวนมาก อาจทำให้ไม่สามารถหาผู้ป่วยได้ตามจำนวนที่ต้องการ และอาจมีผู้ป่วยสูญหายระหว่างการรักษา การแก้ไขคือต้องมีการแนะนำและประชาสัมพันธ์ที่ดี สร้างแรงจูงใจให้กับผู้ป่วย มีการตรวจติดตามและโทรศัพท์สอบถามต่อเนื่อง

15. **ระยะเวลาที่ใช้ในการวิจัยตั้งแต่เริ่มต้นจนสิ้นสุดโครงการ** (Timeline)   ระยะเวลา 1 ปี

16. **สถานที่ทำวิจัย** (Venue of the Study)

หน่วยโรคระบบทางเดินอาหารและตับ ภาควิชาอายุรศาสตร์ คณะแพทยศาสตร์ จุฬาลงกรณ์มหาวิทยาลัย ตึกภูมิสิริมังคลานุสรณ์ ชั้น 2 เลขที่ 1873 ถนน พระรามที่ 4 แขวงปทุมวัน เขตปทุมวัน กรุงเทพมหานคร 10330

17. **การบริหารงานวิจัยและตารางการปฏิบัติงาน** (Tabulation of Research Activities and Timeline)

Activity	2017					2018									
	8	9	10	11	12	1	2	3	4	5	6	7	8	9	10
1. Proposal and IRB															
2. Study period															
3. Data analysis															
4. Conclusion															

18. **งบประมาณ** (Budgets)

**รายการ**	**งบประมาณ**
**หมวดค่าใช้สอย**	
1. ค่าตรวจเลือด TGF-β_1 , TIMP-1, MMP-9, P3NP	200,000 บาท
2. ค่ายาวิตามินดี	10,000 บาท
3. ค่ายาหลอก	3,000 บาท
4. ค่าเจาะเลือดวิตามินดี	80,000 บาท
**รวมเงินทั้งสิ้น**	**293,000 บาท**

19. เอกสารอ้างอิง (References)

1. Lauer GM, Walker BD. Hepatitis C virus infection. The New England journal of medicine. 2001;345(1):41-52. Epub 2001/07/07.
2. Shiffman ML. Natural history and risk factors for progression of hepatitis C virus disease and development of hepatocellular cancer before liver transplantation. Liver transplantation: official publication of the American Association for the Study of Liver Diseases and the International Liver Transplantation Society. 2003;9(11):S14-20. Epub 2003/10/31.
3. Bansal MB FS. Sherlock's Disease of the Liver and Biliary System 2011.
4. Di Rosa M, Malaguarnera M, Nicoletti F, Malaguarnera L. Vitamin D3: a helpful immuno-modulator. Immunology. 2011;134(2):123-39. Epub 2011/09/08.
5. Rahman AH, Branch AD. Vitamin D for your patients with chronic hepatitis C? Journal of hepatology. 2013;58(1):184-9. Epub 2012/08/09.
6. Petta S, Camma C, Scazzone C, Tripodo C, Di Marco V, Bono A, et al. Low vitamin D serum level is related to severe fibrosis and low responsiveness to interferon-based therapy in genotype 1 chronic hepatitis C. Hepatology. 2010;51(4):1158-67. Epub 2010/02/18.
7. Terrier B, Carrat F, Geri G, Pol S, Piroth L, Halfon P, et al. Low 25-OH vitamin D serum levels correlate with severe fibrosis in HIV-HCV co-infected patients with chronic hepatitis. Journal of hepatology. 2011;55(4):756-61. Epub 2011/02/22.
8. Abu-Mouch S, Fireman Z, Jarchovsky J, Zeina AR, Assy N. Vitamin D supplementation improves sustained virologic response in chronic hepatitis C (genotype 1)-naive patients. World journal of gastroenterology : WJG. 2011;17(47):5184-90. Epub 2012/01/05.
9. Nimer A, Mouch A. Vitamin D improves viral response in hepatitis C genotype 2-3 naive patients. World journal of gastroenterology : WJG. 2012;18(8):800-5. Epub 2012/03/01.
10. Abramovitch S, Dahan-Bachar L, Sharvit E, Weisman Y, Ben Tov A, Brazowski E, et al. Vitamin D inhibits proliferation and profibrotic marker expression in hepatic stellate cells and decreases thioacetamide-induced liver fibrosis in rats. Gut. 2011;60(12):1728-37. Epub 2011/08/06.
11. Potter JJ, Liu X, Koteish A, Mezey E. 1,25-dihydroxyvitamin D3 and its nuclear receptor repress human alpha1 (I) collagen expression and type I collagen formation. Liver international : official journal of the International Association for the Study of the Liver. 2013;33(5):677-86. Epub 2013/02/19.
12. Komolmit P, Kimtrakool S, Suksawatamnuay S, Thanapirom K, Chattrasophon K, et al. Vitamin D supplementation improves serum markers associated with hepatic fibrogenesis in chronic hepatitis C patients: A randomized, double-blind, placebo-controlled study. Sci Rep. 2017 Aug 21;7(1):8905

13. Spengler U, Nattermann J. Immunopathogenesis in hepatitis C virus cirrhosis. Clin Sci (Lond). 2007;112(3):141-55. Epub 2007/01/04.

14. Bataller R, Brenner DA. Liver fibrosis. The Journal of clinical investigation. 2005;115(2):209-18. Epub 2005/02/04.

15. Blobe GC, Schiemann WP, Lodish HF. Role of transforming growth factor beta in human disease. The New England journal of medicine. 2000;342(18):1350-8. Epub 2000/05/04.

16. Kanzler S, Baumann M, Schirmacher P, Dries V, Bayer E, Gerken G, et al. Prediction of progressive liver fibrosis in hepatitis C infection by serum and tissue levels of transforming growth factor-beta. Journal of viral hepatitis. 2001;8(6):430-7. Epub 2001/11/13.

17. Tsushima H, Kawata S, Tamura S, Ito N, Shirai Y, Kiso S, et al. Reduced plasma transforming growth factor-beta1 levels in patients with chronic hepatitis C after interferon-alpha therapy: association with regression of hepatic fibrosis. Journal of hepatology. 1999;30(1):1-7. Epub 1999/02/02.

18. Flisiak R JJ, Lapinski TW, Flisiak I, Prokopowiczi D. Effect of pegylated interferon alpha 2b plus ribavirin treatment on plasma transforming growth factor-b1, metalloproteinase-1, and tissue metalloproteinase inhibitor-1 in patients with chronic hepatitis C. WJG. 2005;11(43):6833-8.

19. Liu T, Wang X, Karsdal MA, Leeming DJ, Genovese F. Molecular serum markers of liver fibrosis. Biomarker insights. 2012;7:105-17. Epub 2012/08/09.

20. Martinez SM, Crespo G, Navasa M, Forns X. Noninvasive assessment of liver fibrosis. Hepatology. 2011;53(1):325-35. Epub 2011/01/22.

21. Cholongitas E, Theocharidou E, Goulis J, Tsochatzis E, Akriviadis E, Burroughs K. Review article: the extra-skeletal effects of vitamin D in chronic hepatitis C infection. Alimentary pharmacology & therapeutics. 2012;35(6):634-46. Epub 2012/02/10.

22. Lange CM, Bojunga J, Ramos-Lopez E, von Wagner M, Hassler A, Vermehren J, et al. Vitamin D deficiency and a CYP27B1-1260 promoter polymorphism are associated with chronic hepatitis C and poor response to interferon-alfa based therapy. Journal of hepatology. 2011;54(5):887-93. Epub 2010/12/15.

23. Baur K, Mertens JC, Schmitt J, Iwata R, Stieger B, Eloranta JJ, et al. Combined effect of 25-OH vitamin D plasma levels and genetic vitamin D receptor (NR 1I1) variants on fibrosis progression rate in HCV patients. Liver international : official journal of the International Association for the Study of the Liver. 2012;32(4):635-43. Epub 2011/12/14.

24. Autier P, Gandini S. Vitamin D supplementation and total mortality: a meta-analysis of randomized controlled trials. Archives of internal medicine. 2007;167(16):1730-7. Epub 2007/09/12.

25. Kitson MT, Roberts SK. D-livering the message: the importance of vitamin D status in chronic liver disease. Journal of hepatology. 2012;57(4):897-909. Epub 2012/05/29.

26. Bitetto D, Fabris C, Fornasiere E, Pipan C, Fumolo E, Cussigh A, et al. Vitamin D supplementation improves response to antiviral treatment for recurrent hepatitis C. Transplant international : official journal of the European Society for Organ Transplantation. 2011;24(1):43-50. Epub 2010/07/24.

27. Beinhardt S, Aberle JH, Strasser M, Dulic-Lakovic E, Maieron A, Kreil A, et al. Serum level of IP-10 increases predictive value of IL28B polymorphisms for spontaneous clearance of acute HCV infection. Gastroenterology. 2012;142(1):78-85 e2. Epub 2011/12/24.

28. Marek B, Kajdaniuk D, Mazurek U, Janczewska-Kazek E, Kos-Kudla B, Strzalka B, et al. TGF-beta1 mRNA expression in liver biopsy specimens and TGF-beta1 serum levels in patients with chronic hepatitis C before and after antiviral therapy. Journal of clinical pharmacy and therapeutics. 2005;30(3):271-7.

Information for participant

ข้อมูลสำหรับผู้เข้าร่วมวิจัย

ชื่อโครงการวิจัย ผลของการแก้ไขภาวะขาดวิตามินดีในผู้ป่วยไวรัสตับอักเสบซีที่ตอบสนองต่อยาต้านไวรัสกับการเปลี่ยนแปลงของระดับไซโตไคน์และเอ็นไซม์ที่เกี่ยวเนื่องกับการเพิ่มพังผืดในตับ

<u>แพทย์ผู้ทำวิจัย</u>

ชื่อ ผู้ช่วยศาสตราจารย์นายแพทย์ปิยะวัฒน์ โกมลมิศร์

ที่อยู่ สังกัดหน่วยโรคระบบทางเดินอาหารและตับ ภาควิชาอายุรศาสตร์ คณะแพทยศาสตร์ โรงพยาบาลจุฬาลงกรณ์ ตึกภูมิสิริมังคลานุสรณ์ เลขที่ 1873 ถนน พระรามที่ 4 แขวงปทุมวัน เขตปทุมวัน กรุงเทพมหานคร 10330 เบอร์โทรศัพท์ โทร. 02-256-4265 (ในเวลาราชการ)

<u>แพทย์ผู้ร่วมในโครงการวิจัย</u>

ชื่อ นายแพทย์ศุภพัชญ ศรีภูษณาพรรณ

ที่อยู่ สังกัดหน่วยโรคระบบทางเดินอาหารและตับ ภาควิชาอายุรศาสตร์ คณะแพทยศาสตร์ โรงพยาบาลจุฬาลงกรณ์ ตึกภูมิสิริมังคลานุสรณ์ เลขที่ 1873 ถนน พระรามที่ 4 แขวงปทุมวัน เขตปทุมวัน กรุงเทพมหานคร 10330 เบอร์โทรศัพท์โทร. 02-256-4256 (ในเวลาราชการ), 089-9995696 (ตลอด 24 ชั่วโมง)

<u>แหล่งทุนที่ได้รับ</u>

ได้รับแหล่งเงินทุนสนับสนุนจากทางสมาคมแพทย์ระบบทางเดินอาหารแห่งประเทศไทย

เรียน ผู้เข้าร่วมโครงการวิจัยทุกท่าน

ท่านได้รับเชิญให้เข้าร่วมในโครงการวิจัยนี้เนื่องจากท่านได้รับการวินิจฉัยว่าเป็นโรคไวรัสตับอักเสบซีชนิดเรื้อรังที่หายจากการรักษาด้วยยาต้านไวรัส โครงการวิจัยนี้เป็นเพียงการประเมินผลของการได้รับวิตามินดีเสริม

ก่อนที่ท่านจะตัดสินใจเข้าร่วมในการศึกษาวิจัยดังกล่าว ขอให้ท่านอ่านเอกสารฉบับนี้อย่างถี่ถ้วน เพื่อให้ท่านได้ทราบถึงเหตุผลและรายละเอียดของการศึกษาวิจัยในครั้งนี้ หากท่านมีข้อสงสัยใดๆ เพิ่มเติม กรุณาซักถามจากทีมงานของแพทย์ผู้ทำวิจัย หรือแพทย์ผู้ร่วมทำวิจัยซึ่งจะเป็นผู้สามารถตอบคำถามและให้ความกระจ่างแก่ท่านได้

ท่านสามารถขอคำแนะนำในการเข้าร่วมโครงการวิจัยนี้จากครอบครัว เพื่อน หรือแพทย์ประจำตัวของท่านได้ ท่านมีเวลาอย่างเพียงพอในการตัดสินใจโดยอิสระ ถ้าท่านตัดสินใจแล้วว่าจะเข้าร่วมในโครงการวิจัยนี้ ขอให้ท่านลงนามในเอกสารแสดงความยินยอมของโครงการวิจัยนี้

เหตุผลความเป็นมา

การติดเชื้อไวรัสตับอักเสบซีเป็นสาเหตุที่สำคัญชนิดหนึ่งที่ทำให้เกิดภาวะโรคตับเรื้อรัง เมื่อการติดเชื้อไวรัสตับอักเสบซีดำเนินสู่ภาวะเรื้อรังจะกระตุ้นการเกิดพังผืดในตับจากการหลั่งไซโตไคน์และเอ็นไซม์ ที่เกี่ยวข้องกับการสร้างพังผืดที่เพิ่มขึ้น คือ ทีจีเอฟเบต้าวัน , ทีไอเอ็มพีวัน และมีการลดลงของเอ็มเอ็มพีทู, เอ็มเอ็มพีไนน์ โดยถ้ากระบวนการดังกล่าวยังดำเนินต่อไปก็จะส่งผลให้เกิดภาวะตับแข็งได้ในที่สุด

จากการศึกษาที่ผ่านมาพบว่าวิตามินดีสามารถลดการสร้างพังผืดในตับจากการศึกษาในห้องปฏิบัติการได้และการแก้ไขภาวะขาดวิตามินดีสามารถเปลี่ยนแปลงระดับไซโตไคน์และเอ็นไซม์ที่บ่งชี้ว่ามีการลดลงของการสร้างพังผืดในตับของผู้ป่วยไวรัสตับอักเสบซีเรื้อรัง ผู้วิจัยตั้งสมมติฐานว่า การที่วิตามินดีมีส่วนในการลดการเกิดพังผืดในตับได้นั้น ส่วนหนึ่งน่าจะมีกลไกจากการควบคุมการปรับสมดุลของการสร้างไซโตไคน์และเอ็นไซม์ต่างๆที่เกี่ยวเนื่องกับการเกิดพังผืดในตับ ดังนั้นการให้วิตามินดีเสริมในผู้ป่วยไวรัสตับอักเสบซีที่หายขาดจากการรักษาด้วยยาต้านไวรัสแล้วจะช่วยส่งเสริมทำให้เกิดการลดลงของพังผืดในตับได้ดีขึ้น

จึงเป็นที่มาของการศึกษางานวิจัยนี้ที่ต้องการทราบถึงผลของการได้รับวิตามินดีเสริมในผู้ป่วยไวรัสตับอักเสบซีเรื้อรังที่หายจากการรักษาด้วยยาต้านไวรัสว่าจะส่งผลให้เกิดการเปลี่ยนแปลงของระดับตัววัดพังผืดของตับในซีรั่ม (ทีจีเอฟเบต้าวัน, ทีไอเอ็มพีวัน, เอ็มเอ็มพีทู, เอ็มเอ็มพีไนน์) หรือไม่

วัตถุประสงค์ของการศึกษา

โครงการวิจัยนี้เป็นเพียงการประเมินผลของการได้รับวิตามินดีเสริมในผู้ป่วยไวรัสตับอักเสบซีชนิดเรื้อรังที่หายจากการรักษาด้วยยาต้านไวรัสที่มีภาวะขาดวิตามินดีต่อการเปลี่ยนแปลงของระดับทีจีเอฟเบต้าวัน, ทีไอเอ็มพีวัน, เอ็มเอ็มพีทู, เอ็มเอ็มพีไนน์ เมื่อรับประทานต่อเนื่อง 6 สัปดาห์เปรียบเทียบกับกลุ่มควบคุม โดยวิธีการวัดระดับตัววัดพังผืดของตับในซีรั่ม จำนวนผู้เข้าร่วมในโครงการวิจัย คือ 80 ราย ยาที่ใช้ในการศึกษาครั้งนี้มีชื่อว่าวิตามินดี ซึ่งเป็นยาที่ใช้ในการรักษาผู้ป่วยทั่วไปที่มีภาวะขาดวิตามินดีจากสาเหตุอื่นๆ ยกเว้นโรคไตวายเรื้อรัง

วิธีการที่เกี่ยวข้องกับการวิจัย

ข้อมูลต่อไปนี้จะอธิบายถึงการศึกษาวิจัย ในกรณีที่ท่านตัดสินใจเข้าร่วมการวิจัย และได้ลงนามในหนังสือยินยอมเข้าร่วมการวิจัยฉบับนี้

1. ท่านจะได้รับการตรวจเพื่อการวินิจฉัยและรักษาโรคตามวิธีการที่เป็นมาตรฐาน โดยแพทย์ผู้รักษาของท่าน
2. ผู้วิจัยจะทำการเก็บข้อมูลของท่าน (อาการของโรค ผลการตรวจทางห้องปฏิบัติการ วิธีการรักษา ภาวะแทรกซ้อน ผลการรักษา) จากบันทึกทางการแพทย์ของท่านเท่านั้น โดยชื่อของท่านจะถูกบันทึกเป็นอักษรย่อ และข้อมูลทั้งหมดนี้จะถูกเก็บเป็นความลับ
3. ผู้ทำวิจัยจะขอเจาะเลือด ตรวจวัดระดับวิตามินดี ตรวจค่าการทำงานของตับ ตรวจวัดค่าเม็ดเลือดและเกล็ดเลือด ตรวจวัดน้ำตาลในเลือด และตรวจวัดระดับระดับ ทีจีเอฟเบต้าวัน, ทีไอเอ็มพีวัน, เอ็มเอ็มพีทู, เอ็มเอ็มพีไนน์ในซีรั่มเป็นพื้นฐาน โดยเจาะเลือดปริมาณ 20 มิลลิลิตร เพื่อคัดกรองว่าท่านมีคุณสมบัติที่เหมาะสมที่จะเข้าร่วมในการวิจัย

การศึกษาวิจัยนี้ผู้วิจัยจะขอเจาะเลือด เพื่อตรวจวัดระดับวิตามินดี ตรวจค่าการทำงานของตับ แคลเซี่ยม ฟอสเฟต ตรวจวัดค่าเม็ดเลือดและเกล็ดเลือด ตรวจวัดน้ำตาลในเลือดและตรวจวัดระดับทีจีเอฟเบต้าวัน, ทีไอเอ็มพีวัน, เอ็มเอ็มพีทู, เอ็มเอ็มพี

ไนน์ในซีรั่มเป็นพื้นฐาน โดยผู้วิจัยจะขอเจาะเลือดสองหลอดขนาดเล็ก (หลอดละ 10 ซีซี) หรือเท่ากับ 20 ซีซี (4 ช้อนชา

หากท่านมีคุณสมบัติตามเกณฑ์คัดเข้า ท่านจะได้รับเชิญให้มาพบแพทย์ตามวันเวลาที่ผู้ทำวิจัยนัดหมาย คือ ....(วัน/เวลา) เพื่อซักประวัติและตรวจร่างกาย ประเมินผลทางห้องปฏิบัติการต่าง ๆ ให้ยาวิตามินดีเสริมตามระดับความรุนแรง เจาะเลือดอีกครั้งเมื่อสิ้นสุดโครงการวิจัยโดยตลอดระยะเวลาที่ท่านอยู่ในโครงการวิจัย คือ 6 สัปดาห์และมาพบผู้วิจัยหรือผู้ร่วมทำวิจัยทั้งสิ้น 2 ครั้ง โดยอาสาสมัครที่จะเข้าเกณฑ์การวินิจฉัยภาวะพร่อง(ขาด) วิตามินดี คือต้องมีระดับ 25 OH น้อยกว่า 30 นาโนกรัมต่อซีซี หลังจากนั้นอาสาสมัครจะถูกแบ่งเป็นสองกลุ่มคือกลุ่มที่ได้รับวิตามินดีเสริมกับกลุ่มควบคุมซึ่งจะได้รับยาหลอกซึ่งอาสาสมัครทุกรายที่เข้าร่วมโครงการวิจัยจะมีโอกาสได้รับยาหลอก แต่เมื่อสิ้นสุดโครงการวิจัยแล้วอาสาสมัครที่ได้รับยาหลอกจะได้รับวิตามินดีเสริมทุกรายเช่นเดียวกัน

อนึ่ง ในกลุ่มอาสาสมัครที่ได้รับการตรวจพบระดับ 25 OH มากกว่า 30 นาโนกรัมต่อซีซี จะถือว่าไม่มีภาวะพร่อง (ขาด) วิตามินดีและไม่ได้อยู่ในเกณฑ์คัดเข้าการศึกษา ดังนั้นจึงไม่มีความจำเป็นในการให้วิตามินดีเสริม

<u>ความรับผิดชอบของอาสาสมัครผู้เข้าร่วมในโครงการวิจัย</u>

เพื่อให้งานวิจัยนี้ประสบความสำเร็จ ผู้ทำวิจัยใคร่ขอความความร่วมมือจากท่าน โดยจะขอให้ท่านปฏิบัติตามคำแนะนำของผู้ทำวิจัยอย่างเคร่งครัด รวมทั้งแจ้งอาการผิดปกติต่างๆ ที่เกิดขึ้นกับท่านระหว่างที่ท่านเข้าร่วมในโครงการวิจัยให้ผู้ทำวิจัยได้รับทราบ

เพื่อความปลอดภัย ท่านไม่ควรใช้วัคซีน หรือรับประทานยาอื่น จากการจ่ายยาโดยแพทย์อื่นหรือซื้อยาจากร้านขายยา ขอให้ท่านปรึกษาผู้ทำวิจัย ทั้งนี้เนื่องจากวัคซีน หรือยาดังกล่าวอาจมีผลต่อยาวิตามินดีที่ท่านได้รับจากผู้ทำวิจัย ดังนั้นขอให้ท่านแจ้งผู้ทำวิจัยเกี่ยวกับยาที่ท่านได้รับในระหว่างที่ท่านอยู่ในโครงการวิจัย

<u>ความเสี่ยงที่อาจได้รับ</u>

ผู้เข้าร่วมวิจัยอาจได้รับความเสี่ยง เช่น เสียเวลา ไม่สะดวก ไม่สบาย สูญเสียรายได้ จนถึงความเสี่ยงต่อร่างกาย ต่อจิตใจ ความเสี่ยงจากการรับประทานยาทุกชนิดอาจทำให้เกิดอาการไม่พึงประสงค์ได้ทั้งสิ้นไม่มากก็น้อย แพทย์ผู้ทำการวิจัยขอชี้แจงถึงความเสี่ยงและความไม่สบายที่อาจสัมพันธ์กับยาที่ศึกษาทั้งหมดดังนี้

มีข้อมูลที่แสดงว่ายาวิตามินดีอาจมีผลทำให้เกิดอาการอ่อนเพลีย ปวดศีรษะ คลื่นไส้ อาเจียน และระดับแคลเซียมในร่างกายทำให้สูงเพิ่มขึ้น ซึ่งพบได้น้อยมาก รวมถึงอาการข้างเคียงและความไม่สบายที่ยังไม่มีการรายงานด้วย ดังนั้นระหว่างที่ท่านอยู่ในโครงการวิจัยจะมีการติดตามดูแลสุขภาพของท่านอย่างใกล้ชิด

กรุณาแจ้งผู้ทำวิจัยในกรณีที่พบอาการดังกล่าวข้างต้น หรืออาการอื่นๆ ที่พบร่วมด้วย ระหว่างที่อยู่ในโครงการวิจัย ถ้ามีการเปลี่ยนแปลงเกี่ยวกับสุขภาพของท่าน ขอให้ท่านรายงานให้ผู้ทำวิจัยทราบโดยเร็ว

<u>ความเสี่ยงที่ได้รับจากการเจาะเลือด</u>

ท่านมีโอกาสที่จะเกิดอาการเจ็บ เลือดออก ช้ำจากการเจาะเลือด อาการบวมบริเวณที่เจาะเลือดหรือหน้ามืด และโอกาสที่จะเกิดการติดเชื้อบริเวณที่เจาะเลือดพบได้น้อยมาก และจะไม่มีผลกระทบต่อสุขภาพของท่าน จากการเข้าร่วมงานวิจัยนี้

ความเสี่ยงที่ไม่ทราบแน่นอน

ท่านอาจเกิดอาการข้างเคียง หรือความไม่สบาย นอกเหนือจากที่ได้แสดงในเอกสารฉบับนี้ ซึ่งอาการข้างเคียงเหล่านี้เป็นอาการที่ไม่เคยพบมาก่อน เพื่อความปลอดภัยของท่าน ควรแจ้งผู้ทำวิจัยให้ทราบทันทีเมื่อเกิดความผิดปกติใดๆ ที่เกิดขึ้น

หากท่านมีข้อสงสัยใดๆ เกี่ยวกับความเสี่ยงที่อาจได้รับจากการเข้าร่วมในโครงการวิจัย ท่านสามารถสอบถามจากผู้ทำวิจัยได้ตลอดเวลา

หากมีการค้นพบข้อมูลใหม่ๆ ที่อาจมีผลต่อความปลอดภัยของท่านในระหว่างที่ท่านเข้าร่วมในโครงการวิจัย ผู้ทำวิจัยจะแจ้งให้ท่านทราบทันที เพื่อให้ท่านตัดสินใจว่าจะอยู่ในโครงการวิจัยต่อไปหรือจะขอถอนตัวออกจากการวิจัย

การพบแพทย์นอกตารางนัดหมายในกรณีที่เกิดอาการข้างเคียง

หากมีอาการข้างเคียงใดๆ เกิดขึ้นกับท่าน ขอให้ท่านรีบมาพบแพทย์ที่สถานพยาบาลทันที ถึงแม้ว่าจะอยู่นอกตารางการนัดหมาย เพื่อแพทย์จะได้ประเมินอาการข้างเคียงของท่าน และให้การรักษาที่เหมาะสมทันที หากอาการดังกล่าวเป็นผลจากการเข้าร่วมในโครงการวิจัย ท่านจะไม่เสียค่าใช้จ่ายใดๆ

ประโยชน์ที่อาจได้รับ

การเข้าร่วมในโครงการวิจัยนี้อาจจะทำให้ท่านมีสุขภาพที่ดีขึ้น อาจจะช่วยส่งเสริมการฟื้นฟูและลดพังผืดในตับได้หลังจากการรักษาด้วยยาต้านไวรัส แต่ไม่ได้รับรองว่าสุขภาพของท่านจะต้องดีขึ้นหรือความรุนแรงของโรคจะลดลงอย่างแน่นอน

วิธีการและรูปแบบการรักษาอื่น ๆ ซึ่งมีอยู่สำหรับอาสาสมัคร

ท่านไม่จำเป็นต้องเข้าร่วมโครงการวิจัยนี้เพื่อประโยชน์ในการรักษาโรคที่ท่านเป็นอยู่ เนื่องจากมีแนวทางการรักษาอื่นๆ หลายแบบสำหรับรักษาโรคของท่านได้ ดังนั้นจึงควรปรึกษาแนวทางการรักษาวิธีอื่นๆ กับแพทย์ผู้ให้การรักษาท่านก่อนตัดสินใจเข้าร่วมในการวิจัย

ข้อปฏิบัติของท่านขณะที่ร่วมในโครงการวิจัย

ขอให้ท่านปฏิบัติดังนี้

- ขอให้ท่านให้ข้อมูลทางการแพทย์ของท่านทั้งในอดีต และปัจจุบัน แก่ผู้ทำวิจัยด้วยความสัตย์จริง
- ขอให้ท่านแจ้งให้ผู้ทำวิจัยทราบความผิดปกติที่เกิดขึ้นระหว่างที่ท่านร่วมในโครงการวิจัย
- ขอให้ท่านงดการใช้ยาอื่นนอกเหนือจากที่ผู้ทำวิจัยได้จัดให้ รวมถึงการรักษาอื่น ๆ เช่น การรักษาด้วยสมุนไพร การซื้อยาจากร้านขายยา
- ขอให้ท่านแจ้งให้ผู้ทำวิจัยทราบทันที หากท่านได้รับยาอื่นนอกเหนือจากยาที่ใช้ในการศึกษาตลอดระยะเวลาที่ท่านอยู่ในโครงการวิจัย
- ขอให้ท่านนำยาที่ใช้ในการศึกษาของท่านทั้งหมดที่เหลือจากการรับประทานมาให้ผู้ทำวิจัยทุกครั้งที่นัดหมายให้มาพบ

อันตรายที่อาจเกิดขึ้นจากการเข้าร่วมในโครงการวิจัยและความรับผิดชอบของผู้ทำวิจัย/ผู้สนับสนุนการวิจัย

หากพบอันตรายที่เกิดขึ้นจากการวิจัย ท่านจะได้รับการรักษาอย่างเหมาะสมทันที และท่านปฏิบัติตามคำแนะนำของทีมผู้ทำวิจัยแล้ว ผู้ทำวิจัย/ผู้สนับสนุนการวิจัยยินดีจะรับผิดชอบค่าใช้จ่ายในการรักษาพยาบาลของท่าน และการลงนามในเอกสารให้

ความยินยอม ไม่ได้หมายความว่าท่านได้สละสิทธิ์ทางกฎหมายตามปกติที่ท่านพึงมี

ในกรณีที่ท่านได้รับอันตรายใดๆ หรือต้องการข้อมูลเพิ่มเติมที่เกี่ยวข้องกับโครงการวิจัย ท่านสามารถติดต่อกับผู้ทำวิจัยคือ **นายแพทย์ศุภพัชญ ศรีภูษณาพรรณ ที่เบอร์โทรศัพท์ 089-999-5696 ได้ตลอด 24 ชั่วโมง และเบอร์โทรศัพท์ 02-256-4265 ในเวลาราชการ**

<u>ค่าใช้จ่ายของท่านในการเข้าร่วมการวิจัย</u>

ท่านจะได้รับยาวิตามินดีในโครงการวิจัยจากผู้สนับสนุนการวิจัยโดยไม่ต้องเสียค่าใช้จ่าย

(ค่าใช้จ่ายอื่นที่เกี่ยวข้องกับโครงการวิจัย เช่น ค่าธรรมเนียมทางการแพทย์ และ ค่าวิเคราะห์ทางห้องปฏิบัติการ ผู้สนับสนุนการวิจัยจะเป็นผู้รับผิดชอบทั้งหมด รวมทั้งค่าเดินทางตามความถี่ที่ท่านได้มาพบแพทย์)

<u>การเข้าร่วมและการสิ้นสุดการเข้าร่วมโครงการวิจัย</u>

การเข้าร่วมในโครงการวิจัยครั้งนี้เป็นไปโดยความสมัครใจ หากท่านไม่สมัครใจจะเข้าร่วมการศึกษาแล้ว ท่านสามารถถอนตัวได้ตลอดเวลา การขอถอนตัวออกจากโครงการวิจัยจะไม่มีผลต่อการดูแลรักษาโรคของท่านแต่อย่างใด

ผู้ทำวิจัยอาจถอนท่านออกจากการเข้าร่วมการวิจัย เพื่อเหตุผลด้านความปลอดภัยของท่าน หรือเมื่อผู้สนับสนุนการวิจัยยุติการดำเนินงานวิจัย หรือ ในกรณีดังต่อไปนี้

- ท่านไม่สามารถปฏิบัติตามคำแนะนำของผู้ทำวิจัย
- ท่านรับประทานยาที่ไม่อนุญาตให้ใช้ในการศึกษา
- ท่านตั้งครรภ์ระหว่างที่เข้าร่วมโครงการวิจัย
- ท่านเกิดอาการข้างเคียง หรือความผิดปกติของผลทางห้องปฏิบัติการจากการได้รับยาที่ใช้ในการศึกษา
- ท่านแพ้ยาที่ใช้ในการศึกษา
- ท่านต้องการปรับเปลี่ยนการรักษาด้วยยาตัวที่ไม่ได้รับอนุญาตจากการวิจัยครั้งนี้

<u>การปกป้องรักษาข้อมูลความลับของอาสาสมัคร</u>

ข้อมูลที่อาจนำไปสู่การเปิดเผยตัวท่าน จะได้รับการปกปิดและจะไม่เปิดเผยแก่สาธารณชน ในกรณีที่ผลการวิจัยได้รับการตีพิมพ์ ชื่อและที่อยู่ของท่านจะต้องได้รับการปกปิดอยู่เสมอ โดยจะใช้เฉพาะรหัสประจำโครงการวิจัยของท่าน

จากการลงนามยินยอมของท่านผู้ทำวิจัย และผู้สนับสนุนการวิจัยสามารถเข้าไปตรวจสอบบันทึกข้อมูลทางการแพทย์ของท่านได้แม้จะสิ้นสุดโครงการวิจัยแล้วก็ตาม หากท่านต้องการยกเลิกการให้สิทธิ์ดังกล่าว ท่านสามารถแจ้ง หรือเขียนบันทึกขอยกเลิกการให้คำยินยอม โดยส่งไปที่ **นายแพทย์ศุภพัชญ ศรีภูษณาพรรณ** สาขาวิชาโรคระบบทางเดินอาหารและตับ ภาควิชาอายุรศาสตร์ คณะแพทยศาสตร์ โรงพยาบาลจุฬาลงกรณ์ ตึกภูมิสิริมังคลานุสรณ์ เลขที่ 1873 ถนน พระรามที่ 4 แขวงปทุมวัน เขตปทุมวัน กรุงเทพมหานคร 10330 โทร. 02-256-4265

หากท่านขอยกเลิกการให้คำยินยอมหลังจากที่ท่านได้เข้าร่วมโครงการวิจัยแล้ว ข้อมูลส่วนตัวของท่านจะไม่ถูกบันทึกเพิ่มเติม อย่างไรก็ตามข้อมูลอื่นๆ ของท่านอาจถูกนำมาใช้เพื่อประเมินผลการวิจัย และท่านจะไม่สามารถกลับมาเข้าร่วมในโครงการนี้ได้อีก ทั้งนี้เนื่องจากข้อมูลของท่านที่จำเป็นสำหรับใช้เพื่อการวิจัยไม่ได้ถูกบันทึก

จากการลงนามยินยอมของท่านแพทย์ผู้ทำวิจัยสามารถบอกรายละเอียดของท่านที่เกี่ยวกับการเข้าร่วมโครงการวิจัยนี้ให้แก่แพทย์ผู้รักษาท่านได้

การจัดการกับตัวอย่างชีวภาพที่เหลือ

ตัวอย่างชีวภาพที่ได้จากอาสาสมัคร คือ เลือดที่เหลือจากการวิจัย ผู้วิจัยจะจัดการ ดังต่อไปนี้

- ขอเก็บตัวอย่างไว้เพื่องานวิจัยในอนาคตเป็นระยะเวลา 10 ปี โดยจะเรียงเป็นตัวเลขตามลำดับก่อนหลังที่เข้าโครงการวิจัยของอาสาสมัครแต่ละราย ซึ่งจะสอดคล้องตรงกับข้อมูลของอาสาสมัครซึ่งได้จัดเก็บไว้แล้ว โดยชื่อของข้อมูลทั้งหมดนี้จะถูกเก็บเป็นความลับ สถานที่เก็บรักษาเลือดที่เหลือจากการวิจัย คือ ตึกภูมิสิริมังคลานุ-สรณ์ ชั้น 10 โดยผู้ที่สามารถเข้าถึงตัวอย่างเลือดคือเจ้าหน้าที่และแพทย์ผู้อยู่ในโครงการวิจัย ซึ่งงานวิจัยในอนาคตจะได้ผ่านการพิจารณารับรองจากคณะกรรมการจริยธรรมการวิจัยคณะแพทยศาสตร์ จุฬาลงกรณ์มหาวิทยาลัย

สิทธิ์ของผู้เข้าร่วมในโครงการวิจัย

ในฐานะที่ท่านเป็นผู้เข้าร่วมในโครงการวิจัย ท่านจะมีสิทธิ์ดังต่อไปนี้

1. ท่านจะได้รับทราบถึงลักษณะและวัตถุประสงค์ของการวิจัยในครั้งนี้
2. ท่านจะได้รับการอธิบายเกี่ยวกับระเบียบวิธีการของการวิจัยทางการแพทย์ รวมทั้งยาและอุปกรณ์ที่ใช้ในการวิจัยครั้งนี้
3. ท่านจะได้รับการอธิบายถึงความเสี่ยงและความไม่สบายที่จะได้รับจากการวิจัย
4. ท่านจะได้รับการอธิบายถึงประโยชน์ที่ท่านอาจจะได้รับจากการวิจัย
5. ท่านจะได้รับการเปิดเผยถึงทางเลือกในการรักษาด้วยวิธีอื่น ยา หรืออุปกรณ์ซึ่งมีผลดีต่อท่านรวมทั้งประโยชน์และความเสี่ยงที่ท่านอาจได้รับ
6. ท่านจะได้รับทราบแนวทางในการรักษา ในกรณีที่พบโรคแทรกซ้อนภายหลังการเข้าร่วมในโครงการวิจัย
7. ท่านจะมีโอกาสได้ซักถามเกี่ยวกับงานวิจัยหรือขั้นตอนที่เกี่ยวข้องกับงานวิจัย
8. ท่านจะได้รับทราบว่าการยินยอมเข้าร่วมในโครงการวิจัยนี้ ท่านสามารถขอถอนตัวจากโครงการเมื่อไรก็ได้ โดยผู้เข้าร่วมในโครงการวิจัยสามารถขอถอนตัวจากโครงการโดยไม่ได้รับผลกระทบใด ๆ ทั้งสิ้น
9. ท่านจะได้รับเอกสารข้อมูลคำอธิบายสำหรับผู้เข้าร่วมในโครงการวิจัยและสำเนาเอกสารใบยินยอมที่มีทั้งลายเซ็นและวันที่
10. ท่านมีสิทธิ์ในการตัดสินใจว่าจะเข้าร่วมในโครงการวิจัยหรือไม่ก็ได้ โดยปราศจากการใช้อิทธิพลบังคับข่มขู่ หรือการหลอกลวง

หากท่านไม่ได้รับการชดเชยอันควรต่อการบาดเจ็บหรือเจ็บป่วยที่เกิดขึ้นโดยตรงจากการวิจัย หรือท่านไม่ได้รับการปฏิบัติตามที่ปรากฏในเอกสารข้อมูลคำอธิบายสำหรับผู้เข้าร่วมในการวิจัย ท่านสามารถร้องเรียนได้ที่ **คณะกรรมการจริยธรรมการวิจัย คณะแพทยศาสตร์ จุฬาลงกรณ์มหาวิทยาลัย ตึกอานันทมหิดลชั้น 3 โรงพยาบาลจุฬาลงกรณ์ ถนนพระราม 4 ปทุมวัน กรุงเทพฯ 10330 โทร. 0-2256-4493 ต่อ 14, 15 ในเวลาราชการ**

ขอขอบคุณในการร่วมมือของท่านมา ณ ที่นี้

....

Informed consent sheet

Informed consent sheet

ชื่อโครงการวิจัย ผลของการแก้ไขภาวะขาดวิตามินดีในผู้ป่วยไวรัสตับอักเสบซีที่ตอบสนองต่อยาต้านไวรัสกับการเปลี่ยนแปลงของระดับไซโตไคน์และเอ็นไซม์ที่เกี่ยวเนื่องกับการเพิ่มพังผืดในตับ

วันให้คำยินยอม วันที่.............เดือน.......................................พ.ศ...

ข้าพเจ้า นาย/นาง/นางสาว.. ที่อยู่ ....ได้อ่านรายละเอียดจากเอกสารข้อมูลสำหรับผู้เข้าร่วมโครงการวิจัยวิจัยที่แนบมาฉบับวันที่.................................. และข้าพเจ้ายินยอมเข้าร่วมโครงการวิจัยโดยสมัครใจ

ข้าพเจ้าได้รับสำเนาเอกสารแสดงความยินยอมเข้าร่วมในโครงการวิจัยที่ข้าพเจ้าได้ลงนาม และ วันที่ พร้อมด้วยเอกสารข้อมูลสำหรับผู้เข้าร่วมโครงการวิจัย ทั้งนี้ก่อนที่จะลงนามในใบยินยอมให้ทำการวิจัยนี้ ข้าพเจ้าได้รับการอธิบายจากผู้วิจัยถึงวัตถุประสงค์ของการวิจัย ระยะเวลาของการทำวิจัย วิธีการวิจัย อันตราย หรืออาการที่อาจเกิดขึ้นจากการวิจัย หรือจากยาที่ใช้ รวมทั้งประโยชน์ที่จะเกิดขึ้นจากการวิจัย และแนวทางรักษาโดยวิธีอื่นอย่างละเอียด ข้าพเจ้ามีเวลาและโอกาสเพียงพอในการซักถามข้อสงสัยจนมีความเข้าใจอย่างดีแล้ว โดยผู้วิจัยได้ตอบคำถามต่าง ๆ ด้วยความเต็มใจไม่ปิดบังซ่อนเร้นจนข้าพเจ้าพอใจ

ข้าพเจ้ารับทราบจากผู้วิจัยว่าหากเกิดอันตรายใด ๆ จากการวิจัยดังกล่าว ข้าพเจ้าจะได้รับการรักษาพยาบาลโดยไม่เสียค่าใช้จ่าย

ข้าพเจ้ามีสิทธิที่จะบอกเลิกเข้าร่วมในโครงการวิจัยเมื่อใดก็ได้ โดยไม่จำเป็นต้องแจ้งเหตุผล และการบอกเลิกการเข้าร่วมการวิจัยนี้ จะไม่มีผลต่อการรักษาโรคหรือสิทธิอื่น ๆ ที่ข้าพเจ้าจะพึงได้รับต่อไป

ผู้วิจัยรับรองว่าจะเก็บข้อมูลส่วนตัวของข้าพเจ้าเป็นความลับ และจะเปิดเผยได้เฉพาะเมื่อได้รับการยินยอมจากข้าพเจ้าเท่านั้น บุคคลอื่นในนามของบริษัทผู้สนับสนุนการวิจัย คณะกรรมการพิจารณาจริยธรรมการวิจัยในคน สำนักงานคณะกรรมการอาหารและยาอาจได้รับอนุญาตให้เข้ามาตรวจและประมวลข้อมูลของข้าพเจ้า ทั้งนี้จะต้องกระทำไปเพื่อวัตถุประสงค์เพื่อตรวจสอบความถูกต้องของข้อมูลเท่านั้น โดยการตกลงที่จะเข้าร่วมการศึกษานี้ข้าพเจ้าได้ให้คำยินยอมที่จะให้มีการตรวจสอบข้อมูลประวัติทางการแพทย์ของข้าพเจ้าได้

ผู้วิจัยรับรองว่าจะไม่มีการเก็บข้อมูลใด ๆ เพิ่มเติม หลังจากที่ข้าพเจ้าขอยกเลิกการเข้าร่วมโครงการวิจัยและต้องการให้ทำลายเอกสารและ/หรือ ตัวอย่างที่ใช้ตรวจสอบทั้งหมดที่สามารถสืบค้นถึงตัวข้าพเจ้าได้

ข้าพเจ้าเข้าใจว่า ข้าพเจ้ามีสิทธิ์ที่จะตรวจสอบหรือแก้ไขข้อมูลส่วนตัวของข้าพเจ้าและสามารถยกเลิกการให้สิทธิในการใช้ข้อมูลส่วนตัวของข้าพเจ้าได้ โดยต้องแจ้งให้ผู้วิจัยรับทราบ

ข้าพเจ้าได้ตระหนักว่าข้อมูลในการวิจัยรวมถึงข้อมูลทางการแพทย์ของข้าพเจ้าที่ไม่มีการเปิดเผยชื่อ จะผ่านกระบวนการต่าง ๆ เช่น การเก็บข้อมูล การบันทึกข้อมูลในแบบบันทึกและในคอมพิวเตอร์ การตรวจสอบ การวิเคราะห์ และการรายงานข้อมูลเพื่อวัตถุประสงค์ทางวิชาการ รวมทั้งการใช้ข้อมูลทางการแพทย์ในอนาคตหรือการวิจัยทางด้านเภสัชภัณฑ์เท่านั้น

ข้าพเจ้าได้อ่านข้อความข้างต้นและมีความเข้าใจดีทุกประการแล้ว ยินดีเข้าร่วมในการวิจัยด้วยความเต็มใจ จึงได้ลงนามในเอกสารแสดงความยินยอมนี้

....ลงนามผู้ให้ความยินยอม

(...) ชื่อผู้ยินยอมตัวบรรจง

วันที่ ....เดือน...................................พ.ศ.............................

Case record form

Case number.................................

Date ..../........../............ (DD/MM/YY)

Case Record Form

แบบฟอร์มบันทึกข้อมูลผู้ป่วยโครงการวิจัย

ผลของการแก้ไขภาวะขาดวิตามินดีในผู้ป่วยไวรัสตับอักเสบซีที่ตอบสนองต่อยาต้านไวรัสกับการเปลี่ยนแปลงของระดับไซโตไคน์และเอ็นไซม์ที่เกี่ยวเนื่องกับการเพิ่มพังผืดในตับ

Correction of Vitamin D Deficiency in Chronic Hepatitis C Patients who Had Sustained Virologic Response After Direct-acting Agent Therapy: Effect on Serum Hepatic Fibrogenesis Markers

Baseline Characteristics

Age .... years

Sex ☐ Male ☐ Female

Weight .... kg.

Height .... cm.

BMI kg/m2

Fibroscan .... kpa

MRE ………………..

Ultrasound ………………….

Underlying disease

☐ No ☐ Yes, specify ....

....

Current medications

☐ No ☐ Yes, specify ....

....

HCV diagnosis:

Duration of HCV diagnosis .... months

HCV risk factor(s)

- Heterosexual
- Homosexual
- IVDU
- Blood transfusion
- Tattoo
- Unknown
- Other please define ....

Date of start treatment (DD/MM/YYYY)

............../.............../…….........

Date of end of treatment (DD/MM/YYYY)

............../.............../…….........

Baseline 25(OH) Vitamin D level (ng/mL.)

☐ < 10 ng/mL

☐ 10 -20 ng/mL

☐ 20-30 ng/mL

☐ > 30 ng/mL

After treatment Vitamin D level [illegible] ng/dl

Serology profile

Anti HCV .... HBsAg ....

Anti-HBs .... Anti HIV ....

Baseline HCV genotype before treatment ....

Previous Tx Regimen	Start date	Stop date	VL	Note

Baseline Laboratory

Complete Blood Count

Hct .... Hb........... MCV ....

Wbc ....

% PMN .... % Lymph ....

% Mono .... % Eos. ....

Platelet Count ....

PT .... INR .... PTT ....

Liver Function Test

TB .... DB ....

Albumin .... Globulin ....

SGOT .... SGPT ....

ALP ....

Blood chemistry

BUN .... Cr ....

Na .... K ....

Cl .... CO2 ....

Ca .... Phosphate ....

Others ....

Vitamin D 25(OH)VD

Baseline ....ng/mL

After treatment .... ng/mL

TGF-β1 level

Baseline ....ng/mL

After treatment ....ng/mL

TIMP-1 level

Baseline ....ng/mL

After treatment ....ng/mL

MMP-9 level

Baseline ....ng/mL

After treatment ....ng/mL

P3NP level

Baseline ....ng/mL

After treatment ....ng/mL

Laboratory Follow up

Date (DD/MM/YYYY) ....

Hct .... Hb........... MCV .... Wbc .... % PMN .... % Lymph ....

ANC .... Platelet Count .... PT .... INR .... PTT ....

BUN .... Cr .... TB .... DB .... Albumin .... Globulin ....

SGOT .... SGPT .... ALP ....

Ca .... Phosphate ....

Record by ....

Date ....
