# Supplementary material for: Effect of vitamin D supplementation in patients with chronic hepatitis C after direct-acting antiviral treatment: a randomized, double-blind, placebo-controlled trial
Source: PeerJ. 2021 Feb 9;9:e10709. doi: 10.7717/peerj.10709 (PMC7879942; doi:10.7717/peerj.10709)
Supplement: Supplemental Information 6 [file peerj-09-10709-s006.pdf]

CODEBOOK FOR CATEGORICAL DATA

Variable	Description	Data	Label
**Group**	Randomized group	A<br>B	Vitamin D group<br>Placebo group
**Sex**	Sex	1<br>2	Male<br>Female
**Genotype**	HCV genotype	1<br>3<br>6	Genotype 1<br>Genotype 3<br>Genotype 6
**Cirrhosis**	Presence of cirrhosis	0<br>1	No<br>Yes
